# β-Pix-dependent cellular protrusions propel collective mesoderm migration in the mouse embryo

**DOI:** 10.1038/s41467-020-19889-1

**Published:** 2020-11-27

**Authors:** Tatiana Omelchenko, Alan Hall, Kathryn V. Anderson

**Affiliations:** 1 Developmental Biology Program, Sloan Kettering Institute, Memorial Sloan Kettering Cancer Center, 1275 York Avenue, New York, NY 10065 USA; 2 Cell Biology Program, Sloan Kettering Institute, Memorial Sloan Kettering Cancer Center, 1275 York Avenue, New York, NY 10065 USA

**Keywords:** RHO signalling, Epithelial-mesenchymal transition

## Abstract

Coordinated directional migration of cells in the mesoderm layer of the early embryo is essential for organization of the body plan. Here we show that mesoderm organization in mouse embryos depends on β-Pix (Arhgef7), a guanine nucleotide exchange factor for Rac1 and Cdc42. As early as E7.5, *β-Pix* mutants have an abnormally thick mesoderm layer; later, paraxial mesoderm fails to organize into somites. To define the mechanism of action of β-Pix in vivo, we optimize single-cell live-embryo imaging, cell tracking, and volumetric analysis of individual and groups of mesoderm cells. Use of these methods shows that wild-type cells move in the same direction as their neighbors, whereas adjacent *β-Pix* mutant cells move in random directions. Wild-type mesoderm cells have long polarized filopodia-like protrusions, which are absent in *β-Pix* mutants. The data indicate that β-Pix-dependent cellular protrusions drive and coordinate collective migration of the mesoderm in vivo.

## Abstract (web summary)

Mesodermal directional cell migration is needed to establish body plan but how this is regulated is unclear. Here, the authors show that loss of the guanine nucleotide exchange factor for Rac1 and Cdc42, β-Pix, at mouse gastrulation disrupts the orderly, collective anterior migration of mesoderm cells due to defective cell protrusions.

## Introduction

During gastrulation in the mouse, mesoderm arises continuously from the primitive streak, where individual epiblast cells undergo epithelial-to-mesenchymal transitions. Cells of the nascent mesoderm then migrate anteriorly to destination sites in mesodermal wings^1^, where they then build the organs of the mesoderm and definitive endoderm. Lineage studies have shown that the fate of mesoderm cells depends on the time and location they leave the primitive streak at gastrulation^1^; in addition, transplantation studies have shown that mesoderm cell fate depends on final cell position rather than exclusively on its origin^2^. Classical electron microscopy studies showed that nascent mesoderm cells have cellular protrusions, suggesting that these cells use these protrusions to migrate actively^3^. Despite recent advances in live imaging of post-implantation mouse embryos^4^, it is not known how the nascent mesoderm layer invades between the epithelia of the epiblast and the visceral endoderm, how it migrates long distances to span the circumference of the embryo, or how it then populates specific structures prior to differentiation.

The most complete overview of cell behavior during mesoderm migration in vertebrate embryos comes from zebrafish^5,6^, where cells move towards the anterior midline using both collective migration and cell intercalation, which both depend on non-canonical Wnt/PCP signaling^7,8^. In the mouse embryo, anterior convergence of the mesoderm at somite stages also depends on non-canonical Wnt signaling^6^. However, prior to somite stages, non-canonical Wnt signaling does not appear to be required for migration of mouse mesoderm.

Mouse genetics and live-embryo imaging provide the tools to dissect the cellular and molecular mechanisms of mesoderm migration. Mesoderm behavior is disrupted by mutations in a variety of genes, including signaling molecules (*Fgf8*, *Fgfr1*, *p38Mapk*), transcription factors (e.g. *Eomes*) (reviewed in ref. ^9^), and a larger number of molecules that regulate cell biological processes (e.g. *Nckap1, Rac1*, *RhoA*, *Crumbs2*, *Strip1*)^10–14^. However, the only null mesoderm migration mutant analyzed globally and at cellular resolution is the classical mutant *tw9* (also called *tw18*)^3,15^, which was recently shown to disrupt a scaffolding subunit of the protein phosphatase PP2A^15^. By transmission electron microscopy, the mesoderm in *tw9* mutants lacks the slender filopodia that appear to link neighboring cells in wild-type embryos^3^, consistent with the role of PP2A in cell migration^16,17^.

Recent live imaging studies have also demonstrated functions for the small GTPases RhoA and Rac1 during mesoderm migration out of the primitive streak^13^. Mammalian Rho GTPases and their activators GEFs (guanine nucleotide exchange factors) are central players in the formation of cellular protrusions^18,19^. There are ~80 mammalian GEFs, but the in vivo functions have been determined for only a handful (http://www.informatics.jax.org/)^20^. However, we previously showed that β-Pix (Arhgef7) is essential for early mouse development^21^. β-Pix is a GEF for the Rac1 and Cdc42 GTPases^22,23^ and localizes to focal adhesions^23^. Recent studies indicate that β-Pix both promotes the formation of cellular protrusions and also negatively regulates maturation of focal adhesions in fibroblasts^24^, suggesting that it is a regulator of the dynamic events required for directional cell migration. In a breast cancer mouse model, β-Pix-dependent cellular filopodia-like protrusions are required for lung metastatic colonization by implanted carcinoma cells, indicating a role of β-Pix-mediated protrusions in cancer progression^25,26^.

*β-Pix* null mutant mouse embryos arrest before E8.0 and fail to specify an anterior–posterior body axis because β-Pix is required for collective epithelial migration in the extraembryonic anterior visceral endoderm (AVE) organizer^21^, which produces inhibitors of Wnt and Nodal signaling that restrict the primitive streak to the opposite side of the embryo. In the absence of β-Pix, AVE cells form multiple cellular protrusions due to failure of Cdc42-dependent localization of Rac1 activity. Collective migration of this epithelial population then fails, Wnt and Nodal signaling are not localized, and a proper anterior–posterior body axis does not form^21^.

In addition to collective epithelial migration, three-dimensional collective migration can also take place within tissues^27^ and appears to be particularly important in tumor progression. Here we show that the β-Pix GEF is required in vivo after axis specification for collective migration of a mesenchymal population: the nascent mesoderm. We use optimized high-resolution in vivo live-embryo confocal imaging to directly visualize the movement of cells of the nascent mesoderm to follow the dynamics of cell protrusions and cell movement in vivo. By combining labeling of cells with membrane-GFP and volumetric 3D image analysis, we were able to quantify the migration and protrusions of the nascent mesoderm cells in their native 3D environment. In the late bud/early headfold stage (E7.5) embryo, we see that wild-type mesodermal cells exit from the primitive streak, and then the mesoderm moves both anteriorly, to help elongate the anterior–posterior body axis, and distally, to condense near the anterior midline. In the absence of β-Pix, the mesoderm is thickened and disorganized and individual cells move in random directions. Loss of directional tissue flow in β-Pix mutants is associated with disrupted directionality of protrusions of nascent mesoderm cells and the loss of coordinated cell–cell alignment toward the direction of migration. Thus β-Pix-dependent protrusions direct collective migration and the long-distance tissue flow necessary for organization of the mesoderm in vivo.

## Results

### β-Pix regulates mesoderm organization in the early mouse embryo

We previously showed that β-Pix is essential for early mouse development because of a fundamental role in axis specification controlled by directional migration of the AVE epithelium^21^. β-Pix RNA and protein are broadly expressed in the mouse embryo (Supplementary Fig. 1), and we previously demonstrated that β-Pix is required later for normal development, as deletion of the gene in cells of the embryo proper (the epiblast) using *Sox2-Cre*^28^ (*β-PixΔEpi*) led to embryonic arrest at ~E9.0 (ref. ^21^). Here we examined the function of β-Pix cells derived from the epiblast layer.

**Fig. 1:**
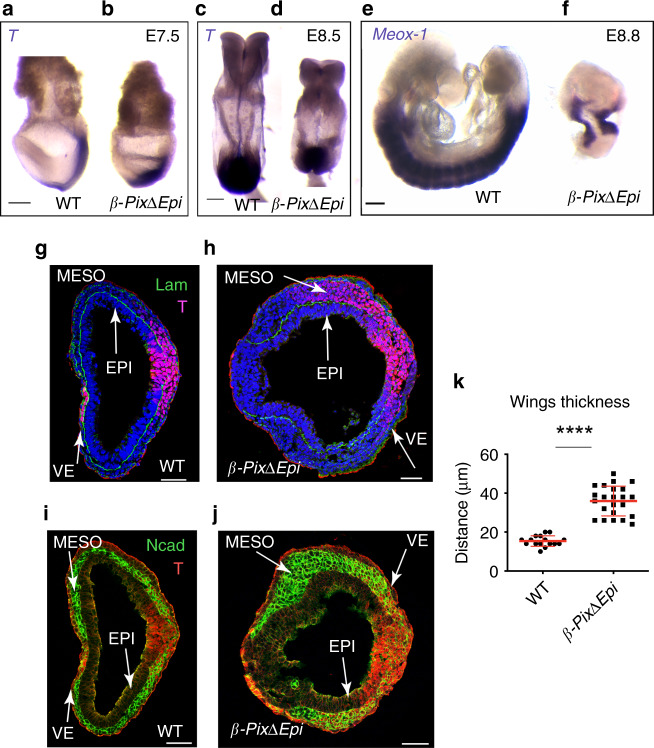
*Arhgef7/β-Pix* is required for mesoderm morphogenesis at gastrulation. **a–f** Phenotypes of *β-PixΔEpi* mutant embryos. In situ hybridization analysis of *Brachyury* (*T*), a marker of the primitive streak, expressed in all nascent mesoderm (**a–d**) showing streak shortening in *β-PixΔEpi* mutant embryos (**b**) as early as late bud stage/early headfold (E7.5); posterior to the right. At E8.5 *β-PixΔEpi* mutant embryo (**d**) shortening is evident; posterior is down. In situ hybridization analysis of *Meox1*, a marker of paraxial mesoderm (**e**, **f**) showing that somites fail to form (**f**) in *β-PixΔEpi* mutant embryos (**g–j**). Transverse single confocal sections of early headfold (E7.5) stage embryos (**g**, **h**); mutants show an expanded primitive streak, marked by the enlarged region of Laminin breakdown and expanded T expression (**h**, **j**). N-cadherin expression marks the mesodermal wings, which are thicker in *β-PixΔEpi* mutant embryos (**j**). **k** Thickness of mesodermal wings in mutant E7.5 embryos is significantly increased, *n* = 16 measurements/4 wild-type embryos, *n* = 23 measurements/6 mutant embryos; mean ± s.d. ****p < 0.0001 by both two-tailed Student’s and one-way ANOVA tests. Scale bars, 150 µm (**a–f**), 50 µm (**g–j**).

At E7.5, *β-PixΔEpi* embryos had a single primitive streak, which appeared to be shorter than in wild type (Fig. 1a, b and Supplementary Fig. 1). *Brachyury* (*T*) was expressed in the primitive streak and axial midline of both wild-type and mutant E8.5 embryos (Fig. 1c, d)^21^. At E8.5–9.0, wild-type embryos had a linear array of segmented somites, marked by expression of *Meox1* (Fig. 1e). At a similar stage, *β-PixΔEpi* embryos were smaller than littermates and expressed *Meox1* in paraxial mesoderm that did not segment into somites (Fig. 1f).

Immunostaining of embryo cryosections showed that epiblast/mesoderm/endoderm germ layer organization was preserved in the absence of β-Pix. T protein was expressed correctly in the primitive streak cells of the posterior epiblast and the nascent mesoderm of wild type and mutant embryos (Fig. 1g, h). The mesoderm marker N-cadherin was expressed specifically in the mesoderm layer of both wild type and mutant embryos (Fig. 1i, j). However, the morphology of the mesoderm wings suggested defects in the behavior of migrating mesoderm: N-cadherin+ mesodermal wings were about twice as thick in *β-PixΔEpi* embryos compared to wild-type (Fig. 1k; 15.4 ± 2.7 µm for wild type, 4 wild-type embryos and 35.6 ± 7.6 µm for mutants, 6 mutant embryos). The wild-type mesoderm layer covers the embryo circumference (Fig. 1i) whereas mutant mesoderm cells do not reach all the way to the anterior of the mutants at this stage (Fig. 1j).

### Wild-type mesoderm cells migrate away from the primitive streak

Beginning at E7.5, the body plan of the mouse embryo begins to take shape, as the embryo elongates in the anterior–posterior axis and mesoderm cells shift toward the future dorsal side of the embryo. The studies described here focused on this later stage of mesodermal wing migration by labeling the entire population of cells with mGFP. We used two complementary live imaging methods, particle image velocimetry (PIV) and single-cell tracking, to characterize the direction and velocity of mesoderm cell movement in the E7.5 mouse embryo. Confocal imaging of the mesoderm in live embryos expressing membrane-GFP (mGFP), using embryos from double-fluorescent reporter mTomato-mGFP (*Rosa26*^*mTmG*^)^29^ females crossed with *Sox2-Cre* males, permitted visualization of the visceral endoderm cells (labeled mTomato), as well as the epiblast cells and the nascent mesoderm cells (both labeled mGFP). By focusing on the mesoderm in the plane of cells between the epiblast and the visceral endoderm, we were able to follow the movement of the most external mesoderm cells (the superficial layer) using confocal microscopy (Supplementary Fig. 2).

**Fig. 2:**
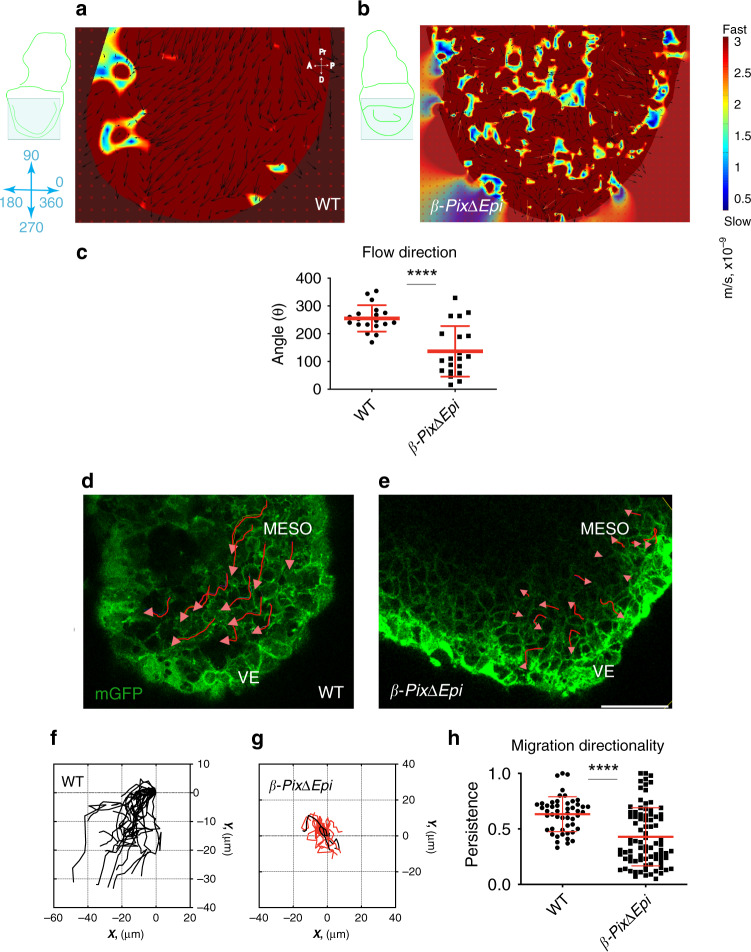
β-Pix is required in vivo for normal migration of the mesoderm wings. **a**, **b** PIV shows defects in global tissue migration in living E7.5 WT and *β-PixΔEpi* mutant embryos. PIV heat maps of velocity magnitude vectors, generated from rendered sagittal confocal time-lapse sections of mesodermal wing of embryos expressing membrane-GFP (mGFP), show directional tissue flow in wild-type embryo (**a**) and disruption of the flow resulting in a patchy pattern in *β-PixΔEpi* mutants (**b**). 0° is right (posterior), 90° is up (proximal), 180° is left (anterior), 270° is down (distal), and 360° is right (posterior). Embryos were positioned in the same orientation (schematics show embryo outline (green), the area of imaging (blue square) and the angles axis in blue). **c** In contrast to the vector angles in the wild type (255° ± 48; *n* = 19, 3 embryos) there is a wider range of vector angles in *β-PixΔEpi* mutants (137° ± 91; *n* = 20, 3 embryos); mean ± s.d. ****p < 0.0001 (two-tailed Student’s *t*-test and one-way ANOVA). **d**, **e** Sagittal confocal time-lapse images of live membrane-GFP embryos show mesodermal wings with overlaid cell migration trajectories (red arrows). **d** Mesoderm cells move distally and anteriorly in the wild-type embryo. In the absence of *β-Pix*, mesoderm cells migrate randomly (**e**). **f, g** Cell migration tracking. Migration of mesoderm cells in wild-type embryo (**f**) is directional and is disrupted in *β-PixΔEpi* (**g**) embryos. **h** Wild-type cells migrate in a persistent way (0.63 ± 0.2; *n* = 50 cells, 4 embryos), which is reduced in *β-Pix* mutant embryos (0.43 ± 0.3; *n* = 86 cells, 6 embryos); mean ± s.d. ***p < 0.0001 (two-tailed Student’s *t*-test), *p* = 0.0043 (one-way ANOVA). Scale bar, 50 µm (**d**, **e**); m/s (**a**, **b**).

PIV is a method to analyze global speed and direction of movements in a tissue from changes in pixel positions (or pixel flow) within time-lapse single-cell-projected images (Fig. 2a, b). In confocal images of membrane-GFP-expressing cells collected over 5 min intervals during 1.5 h of observation time, the distribution of velocity vectors showed that mesoderm tissue flow followed specific trajectories away from the primitive streak (posterior) in wild-type embryos. Tissue in the mesoderm wings moved away from the streak at a distal and anterior angle toward the anterior side (the future head) of the embryo in a predominantly distal direction (towards the future dorsal side of the embryo), at an average angle of 15° anterior to the proximo-distal axis of the embryo (Fig. 2a).

We also tracked the movement of individual membrane-GFP labeled cells. After labeling of all cell membranes in the epiblast and mesoderm with membrane-GFP, using *Rosa26*^*mTmG*^ (ref. ^29^), we followed the behavior of individual mesoderm cells by time-lapse imaging in cultured embryos over longer time (Fig. 2d, e and Supplementary Fig. 2). Individual wild-type mesoderm cells all moved with an overall anterior trajectory during 1.5 h of observation time (Fig. 2d and Supplementary Movie 1), with the cells nearest the streak having the most pronounced anterior movement. Wild-type cells maintained a consistent direction over time; the directional persistence in wild-type cells was 0.63 ± 0.2; *n* = 50 cells, 4 embryos (Fig. 2f, h). Individual cell tracking analysis showed that wild-type cells moved with an average migration speed of 1.2 ± 0.27 µm/min (*n* = 36 cells, 3 embryos). Thus, individual cell movements parallel the global movement of the mesoderm layer identified in the PIV analysis.

### β-Pix is required for directional migration of cells in the embryonic mesoderm

In contrast to the steady anterior movement seen in wild-type mesoderm (Fig. 2a), PIV showed that cell direction was disrupted in *β-PixΔEpi* mutants (Fig. 2b), with patches of adjacent cells appearing to move in different directions. Overall, cells were less oriented relative to the body axis in the mutant than in wild type (Fig. 2c): while the direction of wild-type tissue was consistent (Fig. 2c) 255° ± 48; *n* = 19, 3 embryos), the mutant was much more variable (137° ± 91; *n* = 20, 3 embryos). In addition, patches of mutant cells moved at different velocities, with some patches moving threefold more slowly in the mutant than in wild type.

Tracking the migration of single mesoderm cells for a longer duration also showed that wild-type cells maintained a consistent direction over time (Fig. 2d, f), whereas *β-PixΔEpi* mesoderm cells moved in apparently random directions and lacked persistent directional migration (values < 0.5) (Fig. 2e, g and Supplementary Movie 2). The persistence in *β-PixΔEpi* mutant cells was 0.43 ± 0.3; *n* = 86 cells, 6 embryos (Fig. 2h) as measured for 5 or 6 min intervals between frames for the 1 h 12 min sequence. Thus both PIV and single-cell tracking showed that mesoderm migration in the wild-type E7.5 embryo is directional, and β-Pix is required for persistent directional cell migration of this population of cells.

### β-Pix promotes directional migration of cells in nascent mesoderm explants

**Fig. 3:**
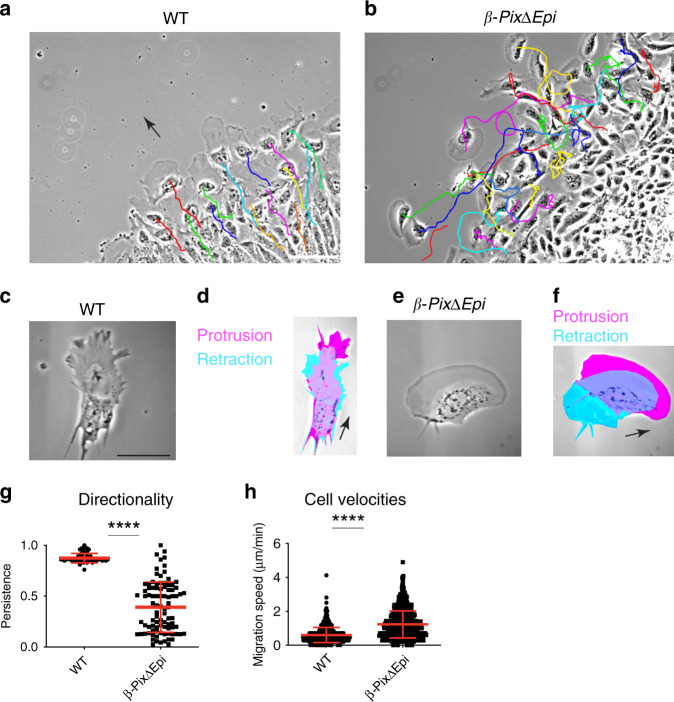
β-Pix regulates migration in mesoderm explants. **a**, **b** Phase contrast time-lapse snapshots with overlaid cell migration trajectories in nascent mesoderm explants imaged for 4 h. Cell tracking shows that migration of wild-type mesoderm explant cells is directional (**a**) and is disrupted in *β-PixΔ* mutant cells (**b**). **c**–**f** Location of cell protrusions depends on β-Pix. Phase contrast images (**c**, **e**) with time-lapse overlays (**d**, **f**) of individual migrating cells on 2D surface. A wild-type mesoderm (**c**, **d**) cell shows protrusions restricted to the leading edge, in contrast to the *β-PixΔ* mutant cell (**e**, **f**), where the entire leading edge protrudes. **g** Persistent directional migration is disrupted in *β-PixΔ* mutant explant cells (0.87 ± 0.05, *n* = 61 wild-type cells, 4 embryos; 0.39 ± 0.2, *n* = 91 mutant cells, 6 embryos); mean ± s.d. ****p < 0.0001 (two-tailed Student’s *t*-test), *p* = 0.0036 (one-way ANOVA). **h** The cell velocities are significantly higher in the absence of *β-Pix* than in wild-type cells. ****p < 0.0001 (two-tailed Student’s *t*-test), *p* = 0.0005 (one-way ANOVA) (0.6 ± 0.45 µm/min, *n* = 641 wild-type cells; 1.23 ± 0.8 µm/min, *n* = 883 mutant cells). Scale bars, 100 µm (**a**, **b**), 30 µm (**c**–**f**).

To compare wild type and mutant cell behaviors at high resolution, we first examined cultured explants of the isolated mesoderm layer^10,30^, which recapitulate, in part, cell movement in vivo. We cultured mesoderm explants dissected from E7.5 mouse embryos plated on fibronectin substrates and imaged cell movements by phase contrast microscopy. Explanted mesoderm layer cells from wild-type E7.5 embryos moved away from the center of the explant towards the free substrate at the edge of the colony (Fig. 3a and Supplementary Movie 3), as previously reported^11^. Individual cells within wild-type explants translocated with steady velocities (0.6 ± 0.45 µm/min, *n* = 641, 3 embryos), whereas cells in mesoderm explants from *β-PixΔEpi* embryos moved more rapidly than wild-type cells (1.2 ± 0.8 µm/min, *n* = 883, 3 embryos).

More striking, while wild-type explant cells moved without cell–cell overlapping or significant neighbor exchange (Supplementary Movie 1), cells in mesoderm explants from *β-PixΔEpi* embryos had intersecting trajectories associated with frequent neighbor exchange (Fig. 3b and Supplementary Movie 4). Mutant cells tumbled, collided, and did not move consistently away from the center of the explant.

During migration, wild-type cells displayed a dominant lamellipodium at the leading edge (Fig. 3c, d and Supplementary Movie 5). In contrast, the protrusion zone of *β-Pix* mutant cells occupied most of the cell perimeter (Fig. 3e-f and Supplementary Movie 6), similar to the behavior of some fish keratocytes^31^. Thus, the data suggest that *β-Pix* restricts the zone of protrusive activity, which allows directional migration of nascent mesoderm cells.

### Polarized β-Pix-dependent protrusions on mesoderm cells contact adjacent epithelial layers

**Fig. 4:**
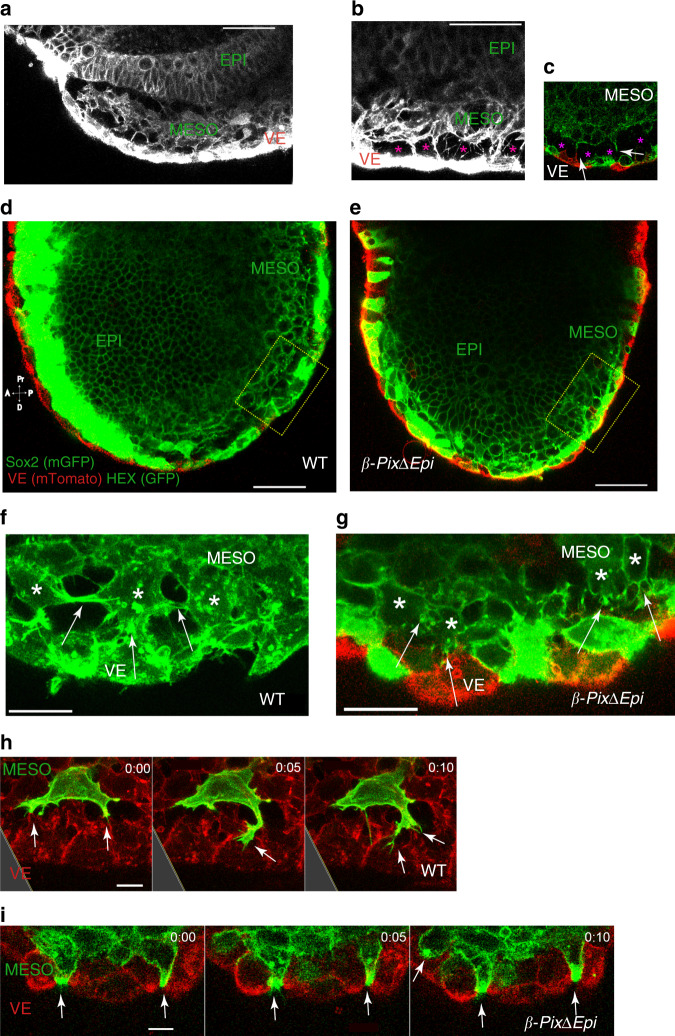
β-Pix is required for directional cellular protrusions in mesoderm cells. **a**, **b** Rendered transverse confocal sections of live embryo expressing mGFP and the visceral endoderm marker Hex-GFP show the mesodermal layer sandwiched between the VE and EPI. **c–e** Rendered sagittal confocal images of live E7.5 embryos expressing mTomato in all VE cells and mGFP in the epiblast (EPI) and mesoderm cells (MESO). Most HEX-GFP cells are on the anterior side. ROI is highlighted. Note cellular protrusions (arrows) emanate from VE and MESO (**c**). Space between VE and MESO labeled with magenta asterisks. **f**, **g** Cellular protrusions (arrows) from mesoderm cells in ROIs from **d**, **e**. **h**, **i** Time-lapse snapshots of cell protrusions (arrows) from wild-type mesoderm cells at the MESO-VE interface demonstrate long, transient protrusions (**e**). Mutant cells at the MESO-VE interface have less dynamic, shorter protrusions with punctate accumulation of mGFP (**f**). Scale bar, 30 µm (**a–e**), 10 µm (**f**, **g**), 5 µm (**h**, **i**). The images are representative and are from *n* > 3 embryos for wild type or mutant genotype (**a–i**). Time scale, h:min.

We then analyzed cellular protrusions in the complex tissue and matrix context present in vivo. Live-embryo imaging showed that wild-type mesoderm is composed of several cell layers, with spaces between the mesoderm and the adjacent tissue layers (Fig. 4a). Time-lapse imaging of transverse sections of mesodermal wings of wild-type E7.5 embryos showed that the space between the mesoderm and the adjacent epithelia was filled with protrusions that contacted both the epiblast and the visceral endoderm layers (Supplementary Movie 7). High-resolution time-lapse imaging of the interface between the mesoderm and the endoderm showed dynamic filopodia-like protrusions formed by superficial mesoderm cells touching both the epiblast and the visceral endoderm epithelia (Fig. 4b, c, d and Supplementary Movie 8).

In contrast to wild-type, cells in the *β-PixΔEpi* mesoderm were tightly packed (Fig. 4e, f and Supplementary Movie 9). Protrusions in wild-type cells to neighboring epithelia were thin, filopodia-like and highly dynamic (Fig. 4g and Supplementary Movie 10). In contrast, mutant protrusions to the adjacent visceral endoderm were shorter and wider (Fig. 4f and Supplementary Movie 11). Mutant protrusions were rich in membrane-GFP vesicles that filled the protrusion (Fig. 4h). Protrusion direction measured in projected images showed a significantly wider distribution of protrusion vectors in *β-PixΔEpi* mutant cells than in wild-type cells (Supplementary Fig. 3).

### β-Pix controls the morphology of protrusions in the mesoderm layer

**Fig. 5:**
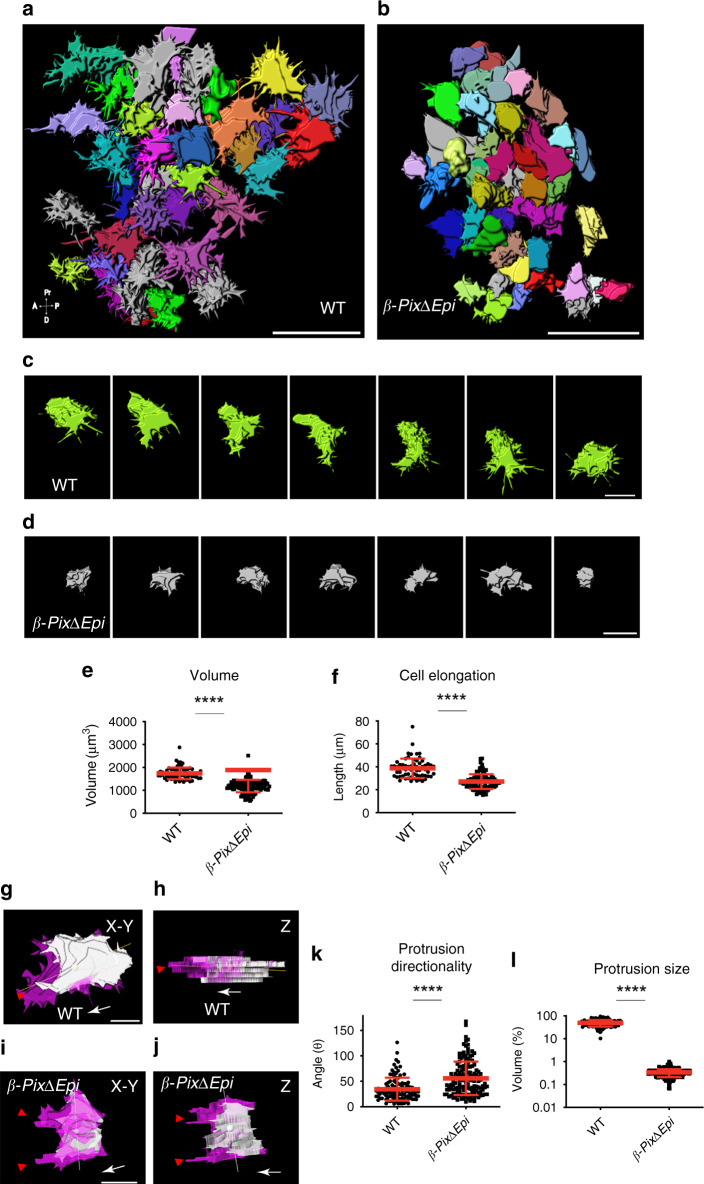
Volumetric analysis of β-Pix-dependent morphologic changes in mesoderm cells. **a**, **b** 3D maps of colored cell surfaces generated from sagittal optical sections of mGFP mesodermal wings’ cells of E7.5 embryos for wild type (**a**) and *β-PixΔEpi* embryos (**b**). Wild-type cells (**a**) show long filopodia-like protrusions that are not present in mutant *β-PixΔEpi* cells (**b**)**. c**, **d** Time-lapse snapshots of individual cells from **a** and **b** demonstrate cell shape and filopodia-like protrusion dynamics in a wild-type cell (**c**), compared to a *β-PixΔ* mutant cell (**d**)**. e** Mutant cells are more compact, with a measured cell volume of 1185 ± 274 µm^3^ (*n* = 134 cells, 7 embryos) in comparison to wild-type 1742 ± 252 µm^3^ (*n* = 76 cells, 6 embryos); mean ± s.d. ****p < 0.0001 (by both two-tailed Student’s *t*-test and one-way ANOVA). **f** The length of the longest axis (38.6 ± 8 µm; *n* = 64 wild-type cells, 6 embryos) is decreased in the mutant (27.8 ± 6 µm; *n* = 106 cells, 7 embryos); mean ± s.d. ****p < 0.0001 (by both two-tailed Student’s *t*-test and one-way ANOVA). Cell surfaces in *X*–*Y* or *Z*-plane views with protrusions (magenta), generated between 0 and 5 min of imaging, with the longest axis (line) and the cell center of mass (sphere) indicated show that the wild type cell (**g, h**) has a major polarized protrusion (red arrowhead) aligned with the longest axis of the cell. **i**, **j** In *β-PixΔEpi* cells, there are multiple protrusions (red arrowheads), which are not aligned with the longest axis of the cell. **k** Protrusion directionality, measured as angles between the axis of the major protrusion and the longest axis of the cells. There is good alignment in wild-type cells (34 ± 23°), which is disrupted in mutant cells (56 ± 33°) (*n* = 108 wild type; *n* = 144 mutant cells, 3 embryos) mean ± s.d. ****p < 0.0001 (two-tailed Student’s *t*-test), *p* = 0.03 (one-way ANOVA). **l** Protrusion size, normalized to total cell volume in mesodermal wing cells is changed significantly in the absence of *β-Pix* in comparison to wild-type cells (49.4 ± 15.8%, *n* = 174 wild-type protrusions; 0.3 ± 0.13%, *n* = 445 mutant protrusions, 3 embryos), mean ± s.d. ****p < 0.0001 (by both two-tailed Student’s *t*-test and one-way ANOVA). Scale bar, 50 µm (**a**, **b**), 10 µm (**c**, **d**, **g**, **j**).

To analyze the behavior of cells in 3D as they migrate within the mesoderm layer, we created high-resolution surfaces using the membrane-GFP signal in time-lapse sequences for both wild type (Fig. 5a) and *β-PixΔEpi* (Fig. 5b) embryos (Supplementary Fig. 4 and Supplementary Movies 12 and 13). 3D reconstructions of migrating cells rendered from sagittal images of late bud (E7.5) membrane-GFP-expressing embryos revealed that wild-type mesoderm cells spread in the *X*–*Y* direction (parallel to the embryo surface) with highly dynamic long filopodia-like protrusions (Fig. 5c). In contrast, *β-PixΔEpi* cells appeared smaller in diameter, were less spread, and lacked well-defined long protrusions (Fig. 5d–f).

To analyze protrusive activity in mesoderm cells in 3D we developed algorithms for visualizing protrusions and retractions along with the centroid and the longest axis of the cell (“Methods”; Fig. 5g–j, Supplementary Fig. 5). Individual migrating wild-type cells had one polarized major protrusion; additionally, the longest axis of the cell was aligned with the long axis of the protrusion (Fig. 5g, h and Supplementary Movie 18). In contrast, individual *β-PixΔEpi* mutant cells were thickened in the *Z*-plane and had multiple protrusions extending towards the direction of migration (Fig. 5i, j and Supplementary Movie 19).

Quantification showed that the direction of the longest protrusion in wild-type cells extended in the direction of migration, with an average protrusion direction of 34 ± 23° relative to the direction of migration (*n* = 108, 3 embryos) (Supplementary Fig. 5). Cells in *β-PixΔEpi* embryos were relatively elongated along the *Z*-axis (radially) and changed shape during migration, with the longest axis rotating in any direction; the average protrusion direction in mutant cells was at a 56 ± 33° angle (*n* = 144, 3 embryos) relative to the direction of migration, a significant increase compared to wild type (Fig. 5k). Protrusion size was also reduced significantly in *β-PixΔEpi* mutant cells (Fig. 5l). Thus, we conclude that *β-Pix* is required for the morphology and polarization of protrusions of nascent mesoderm cells.

### β-Pix is required for cell alignment and collective mesoderm cell migration

Comparing cell direction over time, wild-type mesoderm cells in the embryo migrated in a single direction within 45 ± 32° relative to initial direction between *t* = 0 min and at *t* = 5 min (*n* = 164, 3 embryos, mean ± s.d.) (Supplementary Movie 14). In contrast, mutant mesoderm cells were more variable in direction and moved at angles of 64 ± 39° at *t* = 5 min compared to *t* = 0 min (*n* = 120, 3 embryos, mean ± s.d.). Mutant cells showed a variety of behaviors over time, including moving in opposite directions, cell tumbling, and migration toward each other in the *Z*-axis (Supplementary Movie 15). Consistent with the 2D migration measurements, persistent 3D migration was disrupted in *β-PixΔEpi* mutant cells; in wild-type, cells migrated in the same direction (0.8 ± 0.01; *n* = 24 wild-type cells, 4 embryos), whereas mutant cells changed direction about half the time (0.5 ± 0.09; *n* = 14 mutant cells, 6 embryos; ****p < 0.0001, mean ± s.d.).

**Fig. 6:**
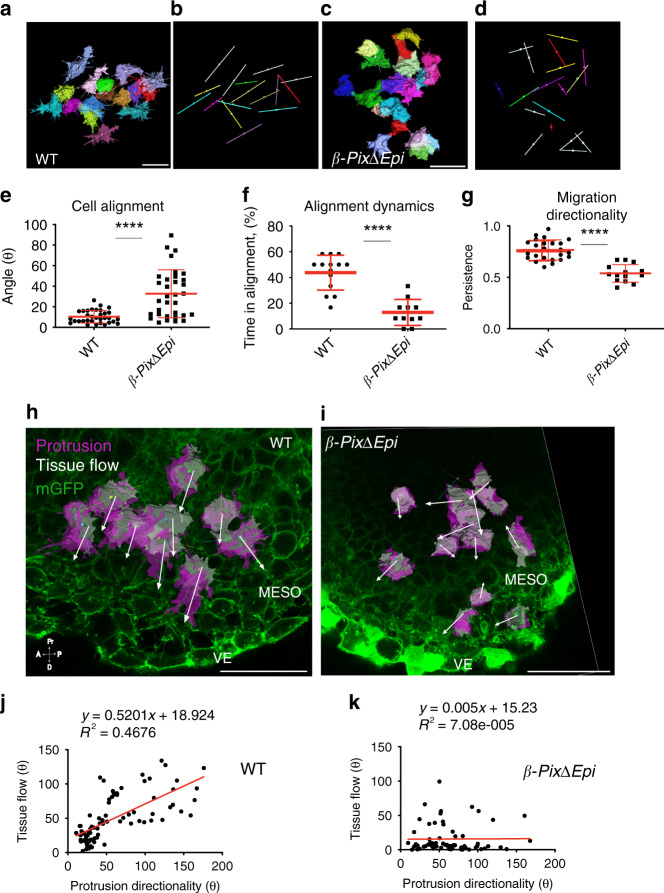
Directional protrusions drive mesenchymal collective tissue flow. **a–f** β-Pix controls cell alignment. **a–d** Longest axes with colored cell surfaces (**a**, **c**) or without them (**b, d**) are generated from membrane-GFP confocal images of mesoderm cells. The longest axis of neighboring mesoderm cells is aligned in wild-type (**a**, **b**) but not in *β-PixΔEpi* mutants (**c**, **d**). **e** Cell alignment is significantly reduced in *β-PixΔEpi* mutant cells (10 ± 6%; *n* = 33 wild-type cells; 33 ± 23%; *n* = 33 mutant cells, 3 embryos); mean ± s.d. ****p < 0.0001 (two-tailed Student’s *t*-test), *p* = 0.03 (one-way ANOVA). **f** Percentage of time cells spend in alignment during collective migration. Wild-type cells are aligned 44 ± 14% of the time (*n* = 14, 3 embryos); mutant cells are aligned 13 ± 10%; time in alignment is significantly reduced in mutant embryos (*n* = 11 mutant cells, 3 embryos); mean ± s.d. ****p < 0.0001 (two-tailed Student’s *t*-test), *p* = 0.01 (one-way ANOVA); angles <60° are considered to be aligned. **g** Directionality of persistent 3D migration is disrupted in *β-PixΔ Epi* mutant cells (0.8 ± 0.01; *n* = 24 wild-type cells, 4 embryos; 0.5 ± 0.09; *n* = 14 mutant cells, 6 embryos; mean ± s.d. ****p < 0.0001 (by both two-tailed Student’s *t*-test and one-way ANOVA). **h**, **i** Time-lapse snapshots at *t* = 0 and *t* = 50 min of 3D images from rendered sagittal sections of living mGFP E7.5 embryos. Maps of selected mesoderm cells are shown with magenta protrusions, longest axes (line) and centroids (sphere); projected 3D displacement vectors (white arrows) are shown on the background of the mGFP embryo. Alignment of protrusions and displacement vectors within multiple rows of cells observed in wild-type embryos (**h**) are not detected in *β-PixΔEpi* mutant embryos (**i**). **j**, **k** Correlation plots graphing protrusion directionality (*X*) and tissue flow angles (*Y*) for 80 wild type and 66 mutant *X*–*Y* pairs show significant correlation coefficient *r* = 0.7 for wild-type cells (**j**) and loss of correlation (*r* = 0.05) in *β-PixΔEpi* mutant cells (**k**). The statistical difference between distributions of **i**, **j** assessed by Chi-squared test of two degrees of freedom was *p* < 10^−8^. Scale bar, 50 µm (**a–d**, **h**, **i**).

To test whether adjacent cells were aligned in 3D within mesodermal wings, we compared the longest axes of neighboring cells. This analysis showed that at least 5–6 cell rows within an optical sagittal section were aligned in wild-type nascent mesoderm (Fig. 6a, b and Supplementary Movie 16), spending at least half of the observation time of 1 h in alignment. In contrast, mutant cells were not aligned (with angles >60°) for ~90% of the 1 h observation time (Fig. 6c–f and Supplementary Movie 17). These data indicate that *β-Pix* is required for cell–cell alignment, a key feature of collective cell migration^27^.

### Dynamic β-Pix-dependent polarized protrusions drive collective migration of the mesoderm

The direction of 3D persistent migration was significantly disrupted in mutants (Fig. 6g). To analyze protrusion directionality and how it correlated with 3D migratory behavior of groups of cells, we visualized protrusions in multiple rows of mesoderm cells (Fig. 6h, i and Supplementary Movie 18). In wild type, the dominant protrusion aligned with both the migratory track and the cell displacement vector (Fig. 6h). Alignment between the direction of the major protrusion and tissue flow was maintained over time in wild-type embryos. There was a significant correlation between protrusion direction and tissue flow in wild type (Fig. 6j; Pearson correlation *r* = 0.7). In contrast, *β-PixΔEpi* mutant cells had disorganized protrusion vectors and disorganized mesodermal tissue flow (Fig. 6i and Supplementary Movie 19), there was no correlation between protrusion direction and tissue flow (Fig. 6k; *r* = 0.008). These data show that β-Pix-dependent protrusions direct tissue flow of the mesodermal wings by regulating cell alignment and drive the unidirectional collective movement needed for organization of the mesoderm.

## Discussion

Our data show that mesoderm tissue organization and movement in the E7.5–8.0 mouse embryo depends on collective cell migration, where cells move together with their neighbors in a concerted anterior direction. High-resolution imaging shows that individual cells have dynamic filopodia-like structures that are polarized towards the leading edge of migrating cells and that neighboring cells move coordinately in the same direction. The migrating cells move through a complex three-dimensional environment with extended filopodia that touch the matrix and neighboring cells, as well as the underlying epiblast and overlying visceral endoderm.

Formation of these filopodia-like structures depends on the GEF β-Pix. In the absence of β-Pix in live embryos, only short stubby cellular protrusions form, and cells do not touch their neighbors with filopodia or move in coordination. β-Pix is an activator of RAC1 and CDC42, and acts by regulating the dynamics of lamellipodia and focal adhesions that allow forward movement of cells^24^. We previously showed that mesoderm migration fails in mouse embryos that lack *Rac1* in epiblast-derived cells^11^. *Rac1* mutant mesoderm cells have defects in both cell migration and adhesion to matrix, whereas the absence of β-Pix in epiblast derivatives does not prevent adhesion, which makes it possible to define specific cellular behaviors in the mutants in vivo. As described in the Saykali et al.^13^ and elsewhere, *T-Cre* (“streak Cre”) reduces but does not eliminate the level of Rho and Cdc42 at the stages of interest, because it is not fully active until ~E7.5. In contrast, our manuscript describes the result of complete elimination of β-Pix in the epiblast and its derivatives. Our study characterizes the migratory behavior in the β-Pix null mutant cells in the mesoderm wings at the stage E7.5. This is a key developmental stage when the body axes are first established, and our data suggest this stage depends on the collective behavior of mesoderm cells.

Explanted β-Pix mutant mesoderm cells show very broad lamellipodia and migrate randomly in a tumbling motion that is very different from the coordinated spreading of wild-type cells. As in explants, β-Pix is required for localized protrusions in vivo, presumably by spatial limitation of the activation of Rac1. In the embryo, we find that the β-Pix-dependent polarized cell protrusions are required for coordination of migration among mesoderm cells. In migrating wild-type cells, fine protrusions are enriched on the side of the cell that is moving forward, whereas protrusions are shorter, wider and localized to all sides of the mutant cells (Fig. 4c, d). The differences between wild-type and mutant cells were most clearly highlighted in the volumetric reconstructions of migrating cells in vivo (Fig. 5).

When tracked over time, adjacent wild-type cells move in the same direction, whereas the orientation of the tracks of adjacent mutant cells are not coordinated, and neighboring cells can even move in opposite directions (Fig. 6g, h). This lack of coordinated cell orientation correlates with differences in global cell flow in the tissue. About 60% of wild-type cells move in the same direction with velocity vector angles less than 40° (compared to the vector at time zero); in contrast, only 30% of mutant cells move in the same direction. Based on the longest axes of cells, 90% of wild-type cells are aligned in parallel, whereas only 36% of mutant cells are aligned with other cells in the field (Fig. 6a–d). Cell movement correlates with cell–cell misalignment: some mutant cells move toward the epiblast in the *Z*-dimension, in contrast to wild-type, cells stay in the same plane of observation (Supplementary Movies 14–15).

β-Pix-dependent mesoderm cell protrusions interact with both the adjacent tissue layers: the visceral endoderm and the epiblast. β-Pix-dependent protrusions could be mediators of long-range intercellular communication, as in the case of protrusions between epithelial somite cells and the ectoderm in chick embryo^32,33^.These protrusions may also coordinate local morphogenetic movements as observed during invagination of the developing mouse eye^34^.

Within the mesoderm layer, β-Pix-dependent collective migration is likely to rely on both contact with the matrix within the mesoderm layer and contact with other mesoderm cells. We suggest that, as in neuronal growth cones^26^, β-Pix-dependent filopodia link focal adhesions to the matrix and to the actin cytoskeleton, and that these links are essential for polarized and collective migration of mesoderm cells.

Phosphorylation of β-Pix on Thr526 can regulate its differential affinity for specific types of extracellular matrix^35^. Thr526 is a target of the isoform of PP2A that includes PPP2R1A, the gene mutated in the mesoderm migration mutant *tw8*. We therefore suggest that β-Pix and PP2A are essential hubs in a protein network that controls collective cell migration through the formation of polarized filopodia. Recent reports on the important roles of RAC1 (ref. ^36^) and β-PIX^37^ in cancer metastasis suggest that the same cell migration mechanisms employed in embryonic development are also crucial for tumor progression. The availability of a set of mouse mutations that disrupt mesoderm migration, together with the methods for analysis of cellular phenotype presented here, provide a foundation for identifying the molecular mechanisms that regulate collective cell migration in vivo.

## Methods

### Mouse strains

Animal experiments were approved by the institutional animal care and use committee (MSKCC, IACUC). Animals were studies in the C3H, CD1 and C57Bl/6 genetic backgrounds. A knockout first allele, *Arhgef7tm1a(EUCOMM)Wtsi*, has a LacZ reporter-tagged insertion^38^. The conditional allele of *Arhgef7* was created by crossing *Arhgef7tm1a(EUCOMM)Wtsi* heterozygous animals with transgenic *FLP* mice to remove the gene-trap cassette by Flp recombinase and leave *loxP* sites^21^. We generated embryos lacking *β-Pix* in the epiblast by crossing *flox/flox* (tm1c allele) females with *β-PixΔ*/*Sox2-Cre* (*β-PixΔEpi*) males. The epiblast specific-expressing Cre line is *Sox2-Cre*^28^. The conditional line was *ROSA mTmG*^29^. The embryos from the *Sox2-Cre* × *mTmG* crosses were labeled VE (mTomato) and Sox2 (mGFP) (Fig. 4 and Supplementary Fig. 2). The mosaic expression Cre line is *EIIA-Cre*^39^. The genotype of the *β-PixΔEpi* (epiblast-deleted) embryos is *Sox2-Cre*/+; *β*-*Pix*^*null/null*^. The conditional allele of *β*-*Pix*^*flox*^ was crossed to *CAG-Cre* (MSKCC Mouse Genetics Core Facilities) to generate the null allele (*β-PixΔ)*. Mosaic embryos expressing both wild-type and null alleles of *β*-*Pix* were obtained by crossing females with *mTmG*; *β*-*Pix*^*flox/flox*^ genotype with *EIIA-Cre*; *β*-*Pix*^*null*^ male. The Hex-GFP line has been described^40^. For timed pregnancies, noon on the day of the vaginal plug was E0.5.

### Genotyping

Mouse ear punches were digested in 100 µl of 300 µg/ml Proteinase K (Roche) in PCR buffer (Invitrogen) overnight at +55 °C, then heat-inactivated. In all, 1–3 µl were used for genotyping. Embryos were digested in 15 µl of Proteinase K/PCR buffer and 5 µl was used for genotyping. Genotyping for *GFP/HEX-GFP, LacZ, Cre*, and *β*-*Pix/Arhgef7* was published^21,41^. Briefly, to detect wild-type β-Pix/Arhgef7, tm1a, tm1b, tm1c, and LoxP, the following primer combinations were used: wild-type β-Pix/Arhgef7 (Arhgef7_40333_F, 5′-TGCTAAAACAGTGGCAGGTG-3′; Arhgef7_40333_R, 5′-ACAGAACACTGCTGCTTCCA-3′); to detect tm1a, primers were Arhgef7_40333_F; CAS_R_Term, 5′-TCGTGGTATCGTTATGCGCC-3′; to detect null allele tm1b (Tm1b_prom_F, 5′-CGGTCGCTACCATTACCAGT-3′; Floxed LR, 5′-ACTGATGGCGAGCTCAGACC-3′); to detect conditional allele tm1c (tm1c_F; tm1c_R, 5′-CCGCCTACTGCGACTATAGAGA-3′); and LoxP (Floxed PNF, 5′-ATCCGGGGGTACCGCGTCGAG-3′; Floxed LR). We used the following primers to detect GFP (Jackson Labs): the forward primer IMR1416: 5′-TCCTTGAAGAAGATGGTGCG-3′ and the reverse primer IMR0872: 5′-AAGTTCATCTGCACCACCG-3′; to detect LacZ, LacZ_forward: 5′-ATCCTCTGCATGGTCAGGTC-3′, LacZ_reverse: 5′-CGTGGCCTGATTCATTC-3′; to detect Cre (Jackson Labs), Crejax1: 5′-TGATGGACATGTTCAGGGATC-3′, Crejax2: 5′-CAGCCACCAGCTTGCATGA-3′.

### LacZ staining and in situ hybridization

For β-galactosidase activity detection, embryos were fixed in 0.2% glutaraldehyde, 2% paraformaldehyde, 5 mM EGTA and 2 mM MgCl_2_ in 0.1 M PBS (pH 7.3) for 10 min. After washing in rinse solution (0.1% sodium deoxycholate, 0.2% IGEPAL, 2 mM MgCl_2_ and 0.1 M PBS (pH 7.3) for 20 min, embryos were incubated in the staining solution consisted of 1 mg/ml X-gal (Lab Scientific) and 5 mM potassium ferri- and ferro-cyanide (Sigma), and 0.4 mM of NBT (4-nitro blue tetrazolium chloride) (Roche), TNBT (5-bromo-4-chloro-3-indoxyl phosphate) (VWR). Whole-mount in situ hybridization was performed on embryos following standard methods^42^. The *Brachyury*, *Meox1* in situ probes were described^43^. Antisense riboprobes were synthesized from vectors available in the lab. Embryos were fixed overnight in 4% paraformaldehyde and processed. The proteinase K treatment was performed at the concentration of 100 μg/ml for 3 min (E6.5 embryos) or 5 min (E7.5 embryos), washed with 2 mg/ml glycine in PBS/0.1% Tween20 and fixed in 4% paraformaldehyde 0.2% glutaraldehyde, and the hybridization was with 200 ng probe at 95 °C for 5 min and overnight at 70 °C in 5× SSC (pH 5), 50% formamide, 500 μg/ml yeast tRNA, 100 μg/ml heparin, 0.5% CHAPS and 0.2% Tween20. The hybridization signal was revealed by using BM Purple (Roche). The embryos were photographed using a stereomicroscope (Leica MZFLIII) equipped with Canon EOS Rebel SL1.

### Immunostaining

Embryos were dissected in ice-cold or room temperature PBS/4% BSA and immunofluorescence staining was performed as described^44^. Immunofluorescence was performed on 4% paraformaldehyde fixed and then frozen sections (Leica Cryostat—15 μm). Sections were stained in PBS with 0.1% Triton and 5% donkey serum. Counterstaining of nuclei was performed with Hoechst (1:40,000). The following primary antibodies were used: anti-*β*-Pix polyclonal rabbit anti-*β*-Pix 07-1450 (Millipore-Chemicon, 1:200), anti-E-cadherin rabbit (Sigma 1:200), anti-N-cadherin rabbit (Cell Signaling 1:200), anti-Laminin rabbit (Sigma 1:500), anti-Brachyury goat (R&D 1:200). Alexa Fluor-conjugated secondary antibodies (Invitrogen) diluted 1:400. Samples were mounted using Vectashield (Vector Labs) mounting media, and slides were imaged with Leica SP5 or SP8 confocal microscopes. Images were analyzed using Imaris (Bitplane Inc). The immunofluorescence data presented in the figures are from multiple embryos (>3).

### Imaging of mouse embryos

Deciduae were dissected from E7.5 pregnant females and placed in ice-cold dissection medium containing phenol red free DMEM/F12 and 10% heat-inactivated fetal bovine serum. Embryos were dissected in the same medium under a stereoscope and cultured in a 1:1 mixture of phenol red free DMEM/F12 and rat serum under 5% CO_2_ and 95% air. For live imaging, dissected embryos were incubated in phenol red free DMEM/F12 supplemented with 50% rat serum in glass bottom dishes (MatTek Corporation) and imaged on Leica SP8 confocal microscopes at 37 °C, 5% CO_2_ in a humidified chamber. Embryos were held in the desired position by making a well into 80% collagen matrix using forceps and placing the embryo inside the well at the very bottom and under the collagen layer. One milliliter 80% collagen was prepared by mixing sterile deionized distilled water (100 µl), 10× DMEM (100 µl), rat collagen (BD Biosciences) (800 µl). To induce polymerization, 25 µl NaOH (filtered 1 N NaOH solution) was added and incubated at 37 °C. High-resolution 1024 × 1024 or 800 × 800, 12-bit Z-stacks of the embryos (pixel size = 0.123 µm (zoom 3×) or 0.223 µm (zoom 1.7×), *Z*-space = 0.5–2 µm) were captured using a HCX PL APO CS ×40 water immersion objective (1.10 NA, Leica). Time-lapse experiments with simultaneous acquisition of multiple channels (bright field, GFP, Tomato) allowed registration for any movement of the embryo during culturing. Only files with no embryo movements were used for analysis. White laser technology (486 nm (GFP signal) and 553 nm (Tomato fluorescent protein signal) and APD high quantum efficiency detectors (Perkin Elmer Avalanche Photo Diode detectors) were used at 0.4–2% laser power. Green and red signals were acquired sequentially. Imaging was performed on embryos at E7.5–E8.2 with unknown genotype obtained from crosses with known genotype of parents, one embryo at a time for 6 h (time interval = 5 or 10 min). For high temporal resolution, 4 h was used (time interval = 2 or 5 min). After imaging, embryos were collected for genotyping.

For optimal time-lapse imaging of gastrulating mouse embryo at single-cell resolution, we used a Leica TCS SP8 X equipped with the pulsed White Light laser source that matches the wavelength of any fluorophore. The confocal microscope was also equipped with Avalanche Photodiodes (APD) designed for extreme low-level light detection. The originality of this approach is the following. In contrast to standard Hybrid Detectors (HyDs)—which can detect most of the fluorescent dyes in visible portion of spectra between 400 and 700 nm but detect only 10–15% of photons at 600 nm—APDs use up to 10-fold less laser power, which is critical for reducing photo toxicity in live mouse embryos. With the hardware setup of pulsed low level, white laser and highly sensitive detectors combined with optimized E7.5 embryo culturing protocol, we imaged early mouse embryos expressing both membrane-GFP and membrane-Tomato reporters at pixel and time resolution allowing characterization of mesodermal wing cell behavior in detail. All time-lapse live-embryo imaging was performed using more than three embryos. The total numbers of embryos and cells examined, for each figure, are summarized in Supplementary Data 1.

### Explant analysis

Primary explant cultures of nascent mesoderm, epiblast, and primitive streak were generated at E7.5 as described^30^. The explants were cultured for 24-48 h on fibronectin-coated glass bottom dishes (MatTek Corporation). Explants were imaged in phase contrast or epifluorescence on inverted Axiovert 200 M equipped with motorized stage and EC Plan-Neofluar ×10 0.3NA Ph1, LD A-Plan ×20 0.3NA Ph1, Plan-Apochromat ×63 1.4NA DIC M27. Images were captured with a Hamamatsu Orca-ER 1394 C4742-80 and controlled by Axiovision software (Zeiss). Explants were kept in normal culture medium (DMEM/Glutamax supplemented with 10% fetal bovine serum (FBS) and 50 μg/ml penicillin, 50 μg/ml streptomycin (P/S) in 5% CO_2_) and 37 °C environment during the acquisition. Regions of the embryos were used for genotyping. Multiple embryos (>3) were used for analysis.

### Morphometric analysis

After genotype confirmation, quantitative morphometric measurements were performed as follows.

Mesoderm thickness was quantified as a distance between the visceral endoderm and the epiblast layers. Number of optical sections between the visceral endoderm and the epiblast in multiple embryos (>3 embryos) were converted to distance in µm and plotted.

Tissue flow was quantified by PIVlab using the Time-Resolved Digital Particle Image Velocimetry Tool for MATLAB (http://pivlab.blogspot.com). Image time-lapse sequences were preprocessed by masking region out of interest for every individual frame. Images were calibrated and analyzed. Every third vector was visualized. Heat map images were generated for 5 min intervals from projected confocal time-lapse images by plotting the velocity magnitude (meters per second)^21^.

For velocity vector angle measurements of the representative images, the velocity vector values *u* and *v* were extracted from PIVlab into Excel and the function *ƒ*_*x*_ = DEGREES(ATAN2(*u*, *v*)) was used to calculate angles in degrees. A positive result represents a counter clockwise angle from the *X*-axis of the image, and a negative value represents a clockwise angle. All angles were converted to 0–360° by adding 360 and the averaged angle was calculated. All angles were normalized by subtracting the average angle value resulting in a linear shift relative to the averaged vector. These normalized velocity vector angles were plotted in a histogram.

Cell tracking of the representative time-lapse images was performed by manually tracking cell centroids over time using MetaMorph (Track Points) and plotted as *X*–*Y* coordinates with starting point at (0;0).

Migration directionality/persistence and speeds were calculated using Metamorph.

### Volumetric single-cell analysis

To extract individual cells in 3D from the mesoderm wings’ optical sectioning files we used the Contour Surface function in Imaris. Sagittal single optical 0.5 µm confocal sections of membrane-GFP embryos are used to outline every individual cell in every section to create a 3D cell surface. By manually drawing the membrane-GFP cell contours on 2D *X*–*Y* optical sections, we focused on cell outlines and protrusions. This manual method allowed for precise outlining of individual cells and their protrusions in all *X*–*Y*–*Z* dimensions from a population of touching cells. Surfaces were created by drawing around an individual cell over time and then repeated for the next cell. To validate a single-cell surface creation accuracy, the surface area of cells over time was measured. The average values for individual cell surface areas over time were differed by ±11%, thus validating the consistency of cell size and accuracy of outlining. The accuracy of contour drawing was confirmed by blinded observers on coded samples. The Surfaces (Imaris) statistics were automatically computed for each surface. Created surfaces were used to perform morphometric measurements and tracking.

The cell surface volume was a measurement of how much space in cubic micrometers a cell surface occupies. The longest axis within a cell surface was defined as following. In Imaris, BoundingBoxOO (object oriented bounding box) was the minimal rectangular cuboid in 3D, which fully encloses an object (a cell surface). BoundingBoxOO length *C* is the Length of the longest principal axis for the best-fitting ellipsoid (in the object oriented bounding box) defined by the three principal axes: *A*, *B*, and *C*. The longest ellipsoid axis (*C*) was used as a measure for the longest cell axis.

Protrusions and retractions in 3D were defined by superimposing two cell surfaces from the two consecutive frames of an individual cell, over a 5-min time interval. Volumes of protrusions were defined as lamellar regions that advanced forward, whereas volumes of retractions were moved away between two frames. Superimposed cell surfaces subtraction allowed us to obtain protrusion volume (marked in magenta), retraction volume (marked in blue), and shared volume (representing the unchanged volume of the cell body, marked in gray). For each volume, a corresponding surface was created.

Protrusion directionality was defined as an angle between the longest axis of the cell and a protrusion vector. A protrusion vector was built by connecting the *X*–*Y*–*Z* coordinates of a cell surface centroid with the *X–Y–Z* coordinates of a protrusion surface centroid.

Rendered cell surfaces were tracked in 3D over time automatically using their centroids. Color-coded in time cell trajectories and displacements were visualized. Cell tracking statistics were exported from Imaris.

Tissue flow in 3D was defined as a set of velocity vectors of individual cells within the tissue, similar to the definition of tissue flow in 2D by PIV. A velocity vector for an individual cell, 1 at time 1, was obtained from two consecutive time frames over a 5-min interval by connecting an *X–Y–Z* coordinate of a cell surface centroid at time 0 min with an *X–Y–Z* coordinate of a cell surface centroid at time 5 min. The next velocity vector for an individual cell, 1 at time 2, was obtained from the next consecutive time frame by connecting an *X–Y–Z* coordinate of a cell surface centroid at time 5 min with an *X–Y-Z* coordinate of a cell surface centroid at time 10 min. An angle between two consecutive velocity vectors was used as a measure of tissue/cell flow. Visualization of the cell centroids and the longest axis of the cell surface (Filament function), protrusions, and retractions (Surface function) were performed using custom MATLAB (v. R2014a, MathWorks) codes within ImarisXT (via Xtension custom algorithms for image processing and segmentation).

Alignment between the major protrusion and the longest axis is measured. Cell alignment was measured as the angle between the longest axes of two pairs of cells over 5 min intervals. Time in alignment was quantified as a percentage of time when cells are in alignment with their neighbors to the total time (60 min). Time in alignment was defined as time when angles between the longest axes of cells relative to the longest axis of the random cell in the group are in the range between 0° and 60°.

### Statistical analysis

Data analysis was performed using Prism 8 or Excel. Statistical analysis was an unpaired two-tailed Student’s *t*-test and one-way nested ANOVA. Details of statistical tests are indicated in the figure legends. Embryos were carefully staged and always imaged at the same developmental stage to ensure compatibility. Kolmogorov-Smirnov, *F*-test, correlation analysis (Pearson coefficient), and Chi square test were performed using statistical computing package R.

### Reporting summary

Further information on research design is available in the Nature Research Reporting Summary linked to this article.

## Supplementary information

Supplementary Information

Reporting Summary

Description of Additional Supplementary Files

Supplementary Data 1

Supplementary Movie 1

Supplementary Movie 2

Supplementary Movie 3

Supplementary Movie 4

Supplementary Movie 5

Supplementary Movie 6

Supplementary Movie 7

Supplementary Movie 8

Supplementary Movie 9

Supplementary Movie 10

Supplementary Movie 11

Supplementary Movie 12

Supplementary Movie 13

Supplementary Movie 14

Supplementary Movie 15

Supplementary Movie 16

Supplementary Movie 17

Supplementary Movie 18

Supplementary Movie 19

## Data Availability

The authors declare that all data supporting the findings of this study are available within the article and its supplementary information files or from the corresponding author upon reasonable request.

## Code availability

Custom MATLAB (v. R2014a, MathWorks) codes within ImarisXT are available at https://github.com/omelchet/Nature-Communication-supplement and https://zenodo.org/record/4071975#.X33whpNKijQ.
