# Supplementary material for: β-Pix-dependent cellular protrusions propel collective mesoderm migration in the mouse embryo
Source: Nat Commun. 2020 Nov 27;11:6066. doi: 10.1038/s41467-020-19889-1 (PMC7695707; doi:10.1038/s41467-020-19889-1)
Supplement: Supplementary file 1 — Supplementary Information [file 41467_2020_19889_MOESM1_ESM.pdf]

Supplementary Figure 1

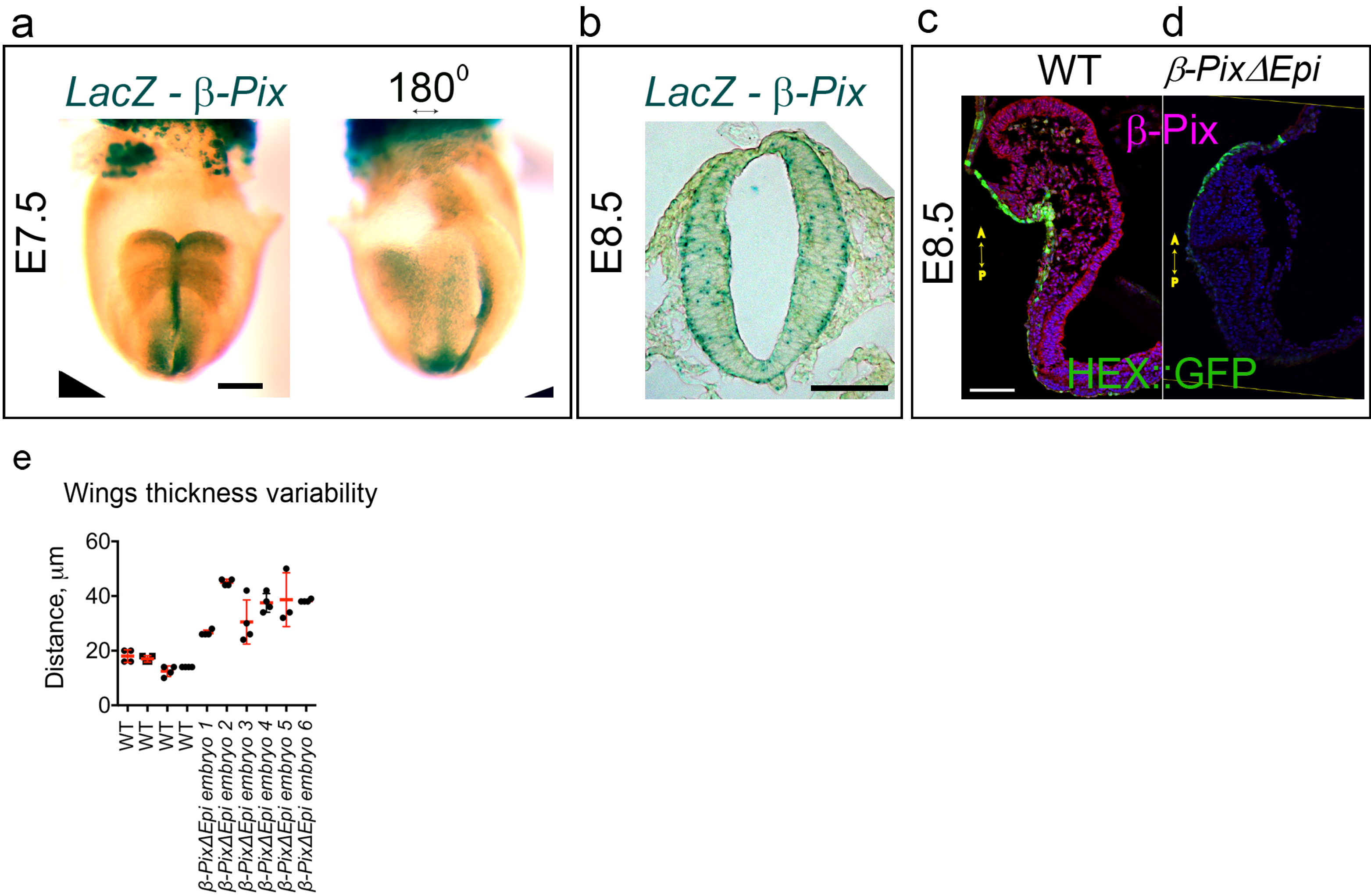

Supplementary Figure 1. β-Pix is expressed in the epiblast and its derivatives and is required for mesoderm morphogenesis

(a-b) Expression patterns of β-Pix at indicated stages of mouse embryo development. β-Galactosidase staining of β-Pix-lacZ (a knockout first allele Arhgef7tm1a(EUCOMM)Wtsi with LacZ reporter-tagged insertion) heterozygotes show strong staining signal in the embryo proper (a). Two different views from the anterior and posterior of the same E7.5 embryo. (b) A cryosection of an E8.5 embryos demonstrates strong signal in the neural tube. (c-d) Immunostaining for β-Pix protein (magenta). Abundant protein expression of β-Pix in the E8.5 wild-type embryo neural plate and mesoderm (c) is abolished in mutant embryo (d). Hex-GFP marks the anterior. (e) Variability between embryos in thickness of mesodermal wings in mutant E7.5 embryos. Related to Figure 1k. n = 16 measurements/ 4 wild-type embryos, n = 23 measurements/ 6 mutant embryos; mean ± s.d. **** is $p < 0.0001$ by both two-tailed Student's and one-way ANOVA tests Scale bars, 150µm (a), 50 µm (b-d).

Supplementary Figure 2

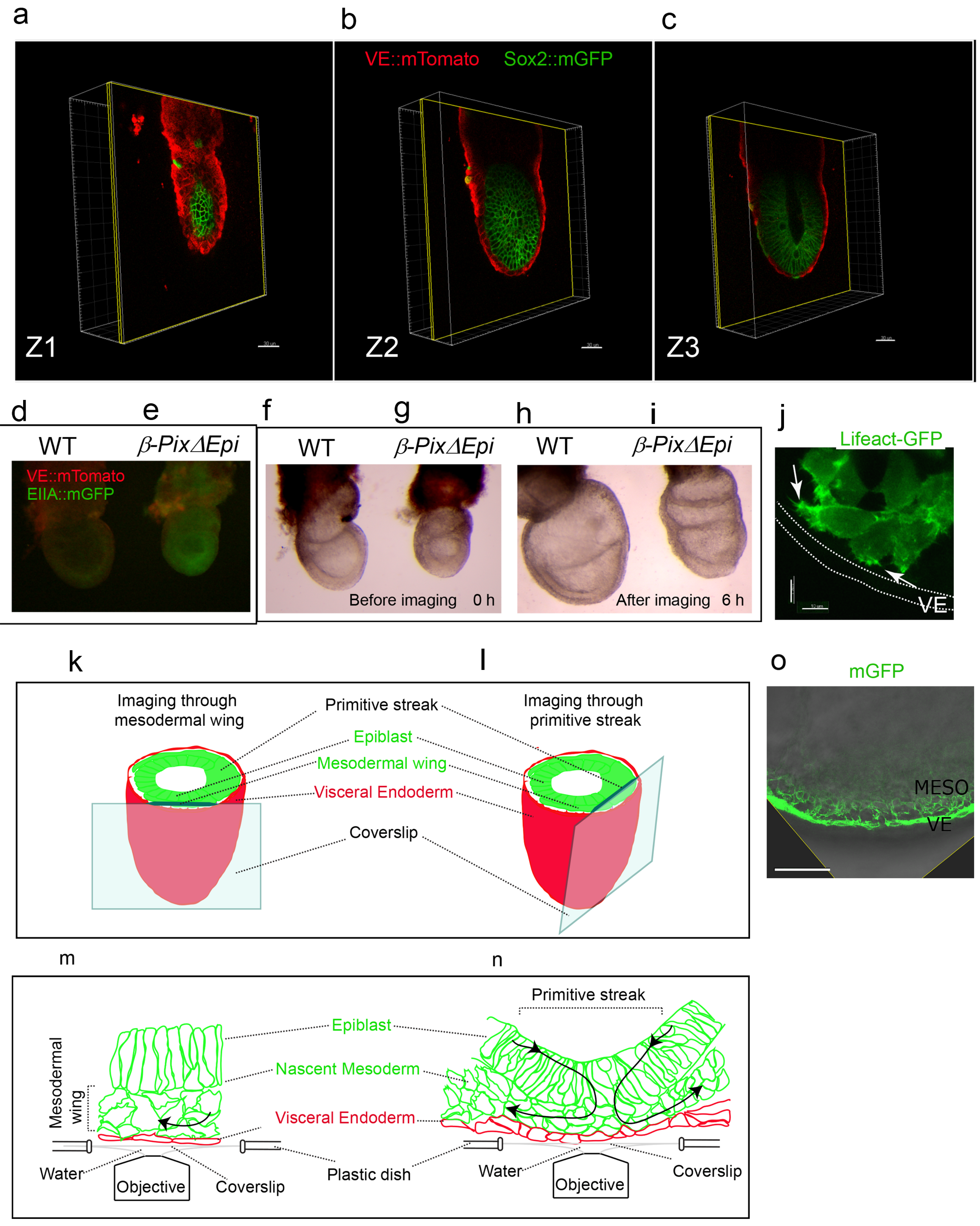

Supplementary Figure 2. Confocal imaging of E7.5 mouse embryos

(a-c) Single confocal sections (Z1-Z3, yellow frames) of live embryos expressing membrane-GFP (mGFP) in the embryo proper and membrane-Tomato (mTomato) in the visceral endoderm (VE). Embryos are from double-fluorescent reporter mTomato-mGFP (Rosa26mTmG) females crossed with Sox2-Cre males. Membrane-GFP cells mark the embryo proper (the epiblast, EPI) and membrane-Tomato cells are the visceral endoderm (VE) epithelial cells. Z1 sections the volume between VE and EPI, the site of nascent mesoderm; Z3 sections through the center of the embryo demonstrating the epiblast with the amniotic cavity. (a-c). (d-e) Freshly dissected live E7.5 wild-type (d) and β-PixΔEpi mutant (e) embryos expressing both mTomato and mGFP imaged at the dissection microscope to visualize fluorescent signal before time-lapse imaging. (f-i) At the end of a 6 h long time-lapse confocal imaging session, both the wild-type and mutant embryos shown in (d-e) have grown and developed. Images are taken using the bright field dissection microscope. (j) Confocal image of cultured live E7.5 wild-type embryo expressing LifeAct-GFP shows normal front-back polarity manifested in polarized accumulation of GFP signal (arrows) in nascent mesoderm cells. (k-m) Schematic of the imaging strategies. (k) Imaging through the mesodermal wings. (l) Imaging through the primitive streak. mTomato-expressing cell layers are in red, mGFP-expressing layers are in green. Dark green line represents optical section. (m-n) Higher resolution cartoon showing the structures of the imaged embryo, the mounting and the microscope objective. mGFP cells are outlined green and mTomato cells are outlined red. Black arrows show direction of tissue flow. (o) Merged image of confocal GFP and bright field channels of the distal end of freshly dissected live E7.5 embryo shows presence of the space between VE and MESO layers. Scale bars, 30 μm (a-c), 10 μm (j), 50 μm (o). The images are representative and are from n > 3 wild-type embryos (a-j, o).

Supplementary Figure 3

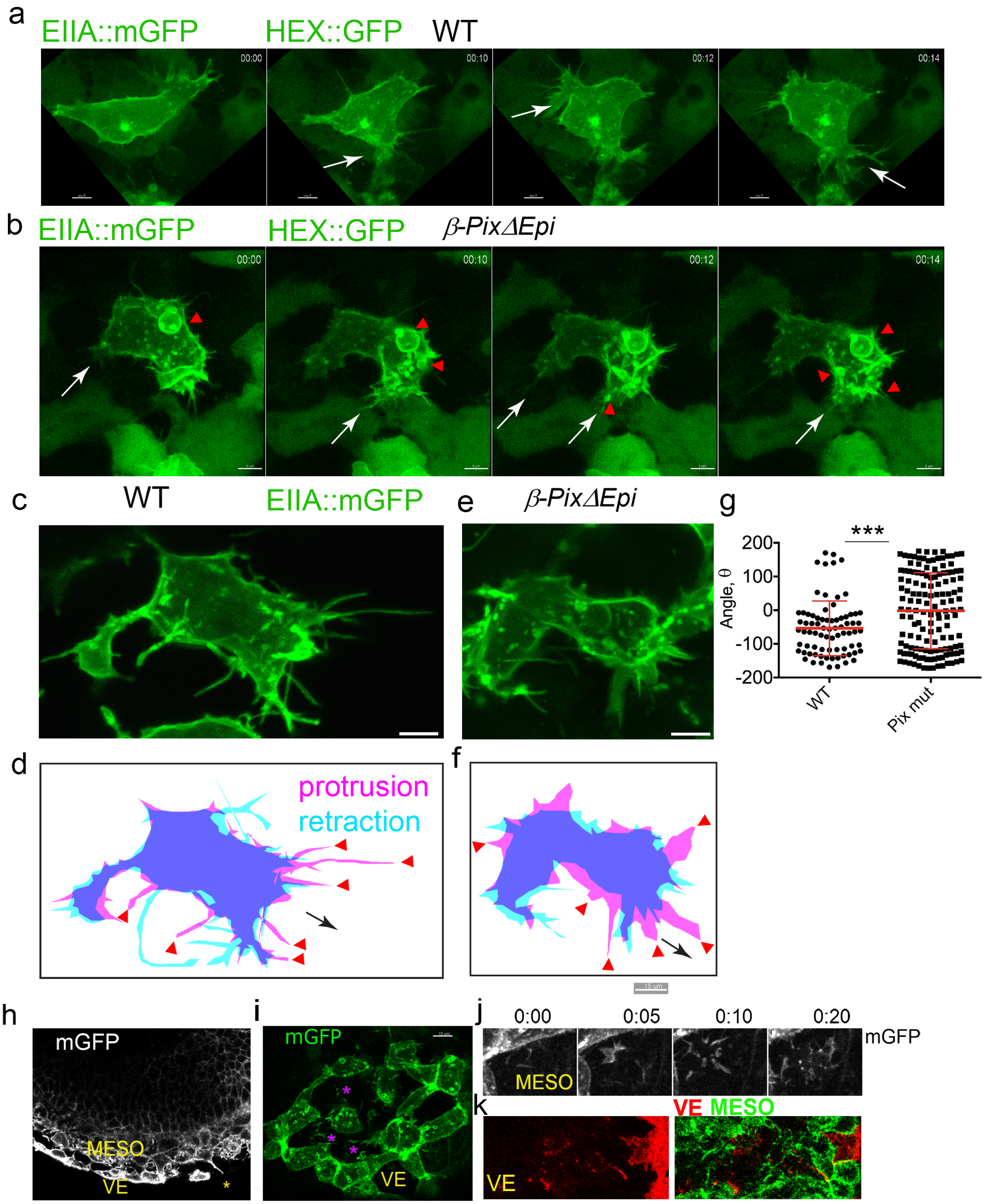

Supplementary Figure 3. β-Pix controls the nature of protrusions of mesoderm cells in vivo

(a-c, e) Rendered sagittal confocal images of live E7.5 embryos mosaically expressing membrane-GFP in mesoderm cells, using the EIIA-Cre system (EIIA-Cre)(ref. 39) and Hex-GFP in the visceral endoderm (VE). (a-b) High-resolution snapshots from the time-lapse sequence. In wild-type (a), mesoderm cells adjacent to VE (with only cytoplasmic GFP) show filopodia-like protrusions (arrows) in contrast to cells with abnormal protrusions (arrows) in β-PixΔEpi mutant embryo. Note accumulation of mGFP vesicles (red arrowheads) (b). (c, e) Confocal images of individual mesoderm cells expressing membrane GFP shows cellular protrusions and their morphology. (d-f) Schematics generated from cells in (c, e) over 5 min time-lapse intervals demonstrate random widened protrusions (red arrowheads) in β-PixΔ mutant cell (d) in contrast to filopodia-like protrusions in wild-type cell (c). Black arrows show direction of cell migration. (g) Directionality of protrusions, measured as an angle relative to the longest axis, is disrupted in β-PixΔ mutant cells. *** is $p = 0.0004$ (two-tailed Student’s) ($-54 \pm 81°$, $n = 77$ protrusions; 3 wild-type embryos; $-2 \pm 10°$, $n = 140$ mutant protrusions; 3 mutant embryos, mean ± s.d.). (h-k) Rendered sagittal (h) and transverse (i-k) confocal images of live E7.5 embryos demonstrate mesoderm protrusions (asterisks) emanating toward the VE. (j) Time-lapse sequence of the mesoderm cell protrusion dynamics at the VE interface. (k) mTomato expressing VE cell protrusions sent toward mGFP mesoderm. Scale bar, 5 μm (a-c, e), 15 μm (h-k). Time scale, h:min. The images are representative and are from $n > 3$ wild-type embryos (a-f, h-k).

Supplementary Figure 4

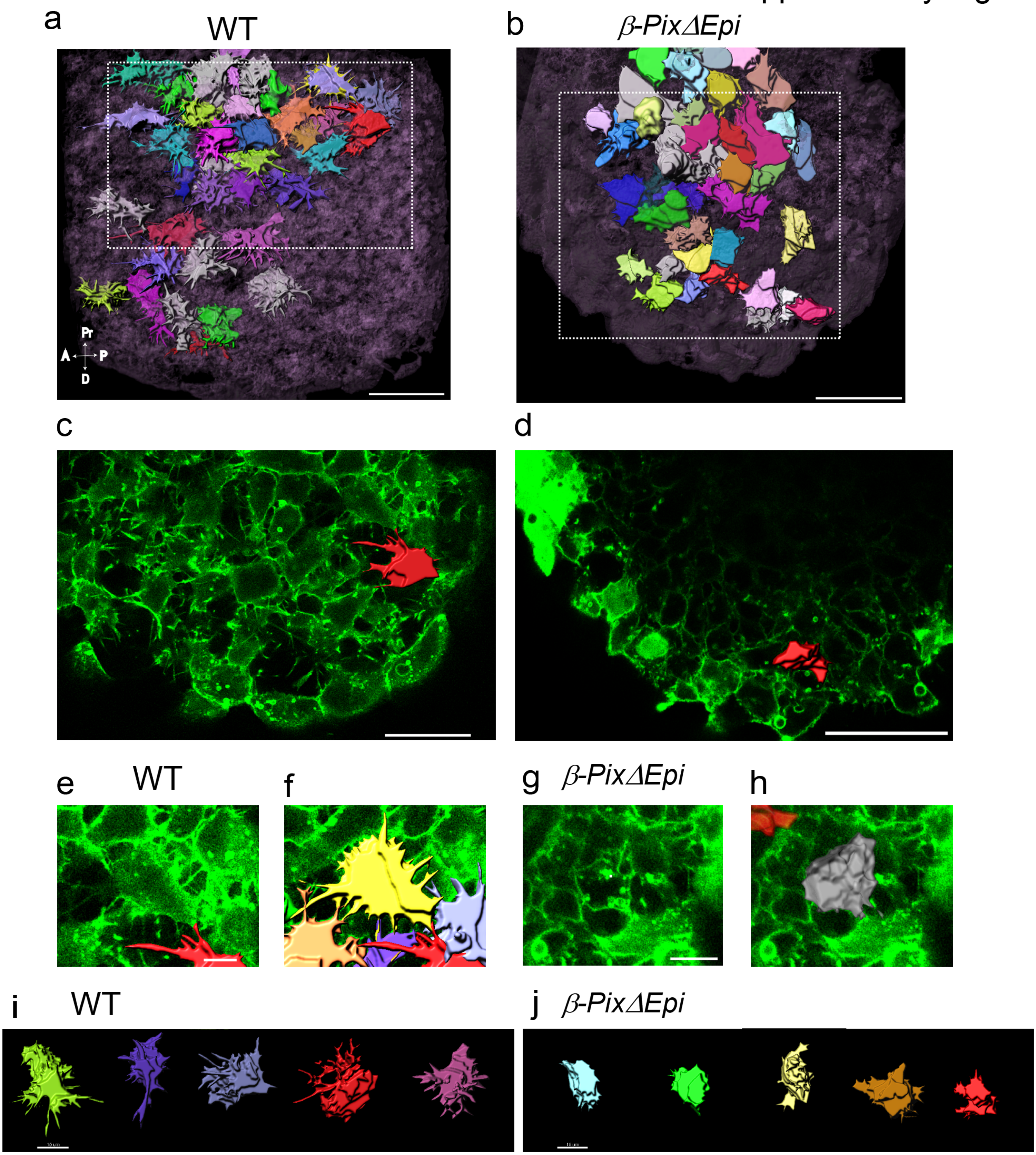

Supplementary Figure 4. 3D cell maps highlight the nature of protrusions of mesoderm cells in vivo
(a-b) 3D embryo maps of colored cell surfaces on the embryo surface (semi-transparent dark magenta) for wild-type (a) and β-PixΔEpi embryos (b). Regions of interest (c-d) demonstrate how sagittal single optical 0.5 μm confocal sections of mGFP embryos are used to outline an individual cell (red) in every section to create a 3D cell surface. (e-h) Individual cells of wild type (e-f) and β-PixΔEpi (g-h) with or without the surface. (i-j) Examples of 3D reconstructed mesoderm cells. (i) Wild-type embryo cells have long filopodia-like protrusions emanating from cell models; in contrast to mutant β-PixΔEpi embryo cells, which lack these structures (j). Scale bars, 50 μm (a-b), 15 μm (c-f).
The images are representative and are from n > 3 embryos per genotype (a-j).

Supplementary Figure 5

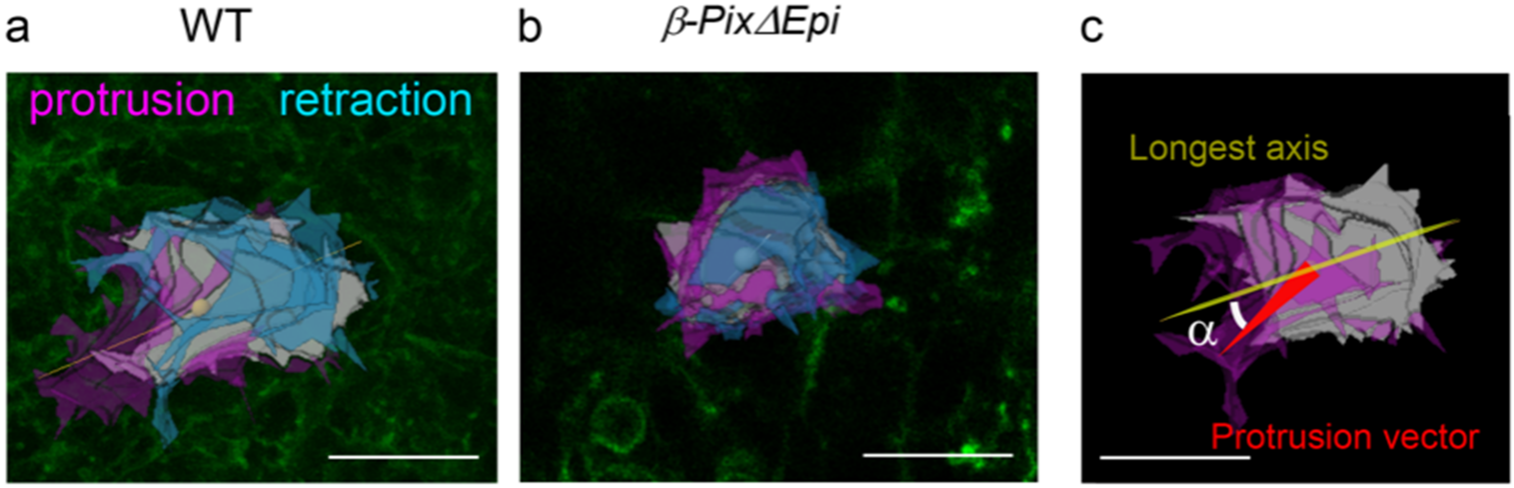

Supplementary Figure 5. Protrusion directionality in 3D cell surface and embryo statistics
(a-b) Three-dimentional reconstructions of live mesoderm wing cells rendered from sagittal confocal images of live E7.5 embryos expressing mGFP. An X-Y view of a shared surface map (gray) of a selected mesoderm cell with magenta highlighted protrusions and blue retraction regions, the longest axis and the centroid. Wild-type cell shows direction protrusion and retraction areas (a) in contrast to nonpolarized ones in mutant (b). (c) Schematic demonstrates measurements of protrusion directionality as angle between the major protrusion and the longest axis. Scale bar, 10 μm (a-c).
