# Supplementary material for: β-Pix-dependent cellular protrusions propel collective mesoderm migration in the mouse embryo
Source: Nat Commun. 2020 Nov 27;11:6066. doi: 10.1038/s41467-020-19889-1 (PMC7695707; doi:10.1038/s41467-020-19889-1)
Supplement: Supplementary file 2 — Reporting Summary [file 41467_2020_19889_MOESM2_ESM.pdf]

nature research

Corresponding author(s): Kathryn V. Anderson

Last updated by author(s): Oct 13, 2020

Reporting Summary

Nature Research wishes to improve the reproducibility of the work that we publish. This form provides structure for consistency and transparency in reporting. For further information on Nature Research policies, see our Editorial Policies and the Editorial Policy Checklist.

Please do not complete any field with "not applicable" or n/a. Refer to the help text for what text to use if an item is not relevant to your study.
For final submission: please carefully check your responses for accuracy; you will not be able to make changes later.

Statistics

For all statistical analyses, confirm that the following items are present in the figure legend, table legend, main text, or Methods section.

n/a	Confirmed	
☐	☒	The exact sample size (*n*) for each experimental group/condition, given as a discrete number and unit of measurement
☐	☒	A statement on whether measurements were taken from distinct samples or whether the same sample was measured repeatedly
☐	☒	The statistical test(s) used AND whether they are one- or two-sided<br>*Only common tests should be described solely by name; describe more complex techniques in the Methods section.*
☒	☐	A description of all covariates tested
☒	☐	A description of any assumptions or corrections, such as tests of normality and adjustment for multiple comparisons
☐	☒	A full description of the statistical parameters including central tendency (e.g. means) or other basic estimates (e.g. regression coefficient) AND variation (e.g. standard deviation) or associated estimates of uncertainty (e.g. confidence intervals)
☐	☒	For null hypothesis testing, the test statistic (e.g. F, t, r) with confidence intervals, effect sizes, degrees of freedom and P value noted<br>*Give P values as exact values whenever suitable.*
☒	☐	For Bayesian analysis, information on the choice of priors and Markov chain Monte Carlo settings
☒	☐	For hierarchical and complex designs, identification of the appropriate level for tests and full reporting of outcomes
☒	☐	Estimates of effect sizes (e.g. Cohen's d, Pearson's r), indicating how they were calculated

Our web collection on statistics for biologists contains articles on many of the points above.

Software and code

Policy information about availability of computer code

Data collection	Data were collected using Axiovision software (www.zeiss.com), LAS X Microscope Imaging Software (www.leica-microsystems.com).
Data analysis	Data were analyzed by the Time-Resolved Digital Particle Image Velocimetry Tool for MATLAB (http://pivlab.blogspot.com), MetaMorph (www.moleculardevices.com), Imaris and custom MATLAB (v. R2014, MathWorks) codes within ImarisXT (via Xtension custom algorithms for image processing and segmentation) that are available at https://github.com/omelchet/Nature-Communication-supplement . Statistical data were analyzed using Excel, Prism and R.

For manuscripts utilizing custom algorithms or software that are central to the research but not yet described in published literature, software must be made available to editors and reviewers. We strongly encourage code deposition in a community repository (e.g. GitHub). See the Nature Research guidelines for submitting code & software for further information.

Data

Policy information about availability of data

All manuscripts must include a data availability statement. This statement should provide the following information, where applicable:
- Accession codes, unique identifiers, or web links for publicly available datasets
- A list of figures that have associated raw data
- A description of any restrictions on data availability

The datasets generated and/or analyzed during the current study are available from the corresponding author on reasonable request.

Field-specific reporting

Please select the one below that is the best fit for your research. If you are not sure, read the appropriate sections before making your selection.

☒ Life sciences ☐ Behavioural & social sciences ☐ Ecological, evolutionary & environmental sciences

Life sciences study design

All studies must disclose on these points even when the disclosure is negative.

Sample size	Sample size was determined based on the number of independent experiments and on the number of available data points from each experiment to reach statistical significance (at least three independent experiments/three embryos from different litters)
Data exclusions	Data from dividing cells of cells with inconsistent over time surface area that was a consequence of poor cell segmentation were excluded from analysis.
Replication	Replication attempts were successful and data were reproducible.
Randomization	Samples were allocated into experimental groups based on the genotype (wild-type versus mutant).
Blinding	Blinding was not possible due to strong/dinstict phenotype in embryos and cells that have been analyzed.

Reporting for specific materials, systems and methods

We require information from authors about some types of materials, experimental systems and methods used in many studies. Here, indicate whether each material, system or method listed is relevant to your study. If you are not sure if a list item applies to your research, read the appropriate section before selecting a response.

Materials & experimental systems

n/a	Involved in the study	
☐	☒	Antibodies
☐	☒	Eukaryotic cell lines
☒	☐	Palaeontology and archaeology
☐	☒	Animals and other organisms
☒	☐	Human research participants
☒	☐	Clinical data
☒	☐	Dual use research of concern

Methods

n/a	Involved in the study	
☒	☐	ChIP-seq
☒	☐	Flow cytometry
☒	☐	MRI-based neuroimaging

Antibodies

Antibodies used	anti-beta-Pix, SH3 domain polyclonal rabbit (07-1450, Millipore-Chemicon), anti-E-cadherin rabbit (3195P, clone 24E10, lot: 10, Cell Signaling), anti-N-cadherin rabbit (13116S, clone D4R1H, lot: 4, Cell Signaling), anti-Laminin rabbit (L-9393, Sigma), anti-Brachyury goat (AF2085, R&D), Alexa Fluor-conjugated secondary antibodies (Invitrogen, A11097, lot: 1871957; A11008, lot: 1761258)
Validation	Each antibody was validated in the lab and published in previous studies and using knockout or siRNA mediated knockdown (PMID: 25512563, PMID: 31371508, PMID: 27870829. Additional information on validation is available from websites of Millipore, Cell signaling, Sigma, R&D and Invitrogen.

Eukaryotic cell lines

Policy information about cell lines

Cell line source(s)	cell lines were not used in this study
Authentication	n/a
Mycoplasma contamination	n/a
Commonly misidentified lines (See ICLAC register)	n/a

Animals and other organisms

Policy information about studies involving animals; ARRIVE guidelines recommended for reporting animal research

Laboratory animals	Animals (mice) were in the C3H, CD1 and C57Bl/6 genetic backgrounds, males and females at their reproductive age (6-8 months old).
Wild animals	n/a
Field-collected samples	n/a
Ethics oversight	This work was approved by the Memorial Sloan Kettering Cancer Center IACUC (protocol number 02-06-013) and studies were conducted in accordance with their guidelines.

Note that full information on the approval of the study protocol must also be provided in the manuscript.

This checklist template is licensed under a Creative Commons Attribution 4.0 International License, which permits use, sharing, adaptation, distribution and reproduction in any medium or format, as long as you give appropriate credit to the original author(s) and the source, provide a link to the Creative Commons license, and indicate if changes were made. The images or other third party material in this article are included in the article's Creative Commons license, unless indicated otherwise in a credit line to the material. If material is not included in the article's Creative Commons license and your intended use is not permitted by statutory regulation or exceeds the permitted use, you will need to obtain permission directly from the copyright holder. To view a copy of this license, visit http://creativecommons.org/licenses/by/4.0/
