# Supplementary material for: β-Pix-dependent cellular protrusions propel collective mesoderm migration in the mouse embryo
Source: Nat Commun. 2020 Nov 27;11:6066. doi: 10.1038/s41467-020-19889-1 (PMC7695707; doi:10.1038/s41467-020-19889-1)
Supplement: Supplementary file 3 — Description of Additional Supplementary Files [file 41467_2020_19889_MOESM3_ESM.pdf]

Title: Supplementary Data 1.

Description: Summary table on the statistics of the wild-type and mutant embryos imaged.

Title: Supplementary Movie 1: Wild-type mesodermal wing cells move coordinately toward the anterior and distal poles of the embryo

Description: Nascent mesoderm cells expressing membrane-GFP were imaged from the embryo's lateral side (sagittal optical section) at E7.5. Cell trajectories are color-coded in time. The anterior side is labeled with Hex-GFP-expressing visceral endoderm cells. Rendered sagittal confocal sections were projected with total 8 □m thickness. Images were taken by time-lapse confocal microscopy acquiring every 5 min for 1 h 30 min.

Title: Supplementary Movie 2: Mutant mesodermal wing cells move with random trajectories

Description: Nascent mesoderm cells expressing membrane-GFP were imaged from the lateral sides of mutant *β-Pix□Epi* embryos (sagittal optical section) at E7.5. Hex-GFP is expressed in anterior visceral endoderm cells. Cell trajectories are color-coded in time. Rendered sagittal confocal sections were projected with total 6 □m thickness. Images were taken by time-lapse confocal microscopy acquiring every 6 min for 1 h 12 min.

Title: Supplementary Movie 3: Wild-type nascent mesoderm cells translocate toward the free substrate at the edge of the explant

Description: Mesoderm migration in explants from E7.5 wild-type embryos is coordinated and directional, with cells moving along aligned tracks (labeled). Mesodermal wings were dissected and plated on the fibronectin-coated glass surface. Time-lapse images were taken by phase-contrast microscopy acquiring every 5 min for 4 h.

Title: Supplementary Movie 4: Cells in mutant mesoderm explants lose orientation

Description: In the absence of β-Pix, cell migration lost its directionality, with cells moving in apparently random tracks (labeled). Mutant cells tumbled, paused and did not coordinate their trajectories with neighboring cells. Time-lapse images were taken by phase-contrast microscopy acquiring every 5 min for 4 h.

Title: Supplementary Movie 5: Localized protrusion dynamics in an explanted wild-type mesoderm cell migrating on the free substrate

Description: A cell with protrusions that occupied ~30% of the cell's periphery at the leading edge. Time-lapse images were taken by phase-contrast microscopy acquiring every 1 min for 0.5 h.

Title: Supplementary Movie 6: Protrusion dynamics are expanded in a mutant nascent mesoderm cell migrating on the free substrate

Description: A mutant cell with a broad protrusion occupying more than 50% of the cell's periphery migrates with turns. Time-lapse images were taken by phase-contrast microscopy acquiring every 1 min for 0.5 h.

Title: Supplementary Movie 7: In wild-type embryos the space between the mesoderm and the adjacent epithelia is filled with protrusions contacting both the epiblast and the endoderm layers

Description: Imaging was done of optical transverse sections at the anterior of the mesodermal wings of wild-type E7.5 embryos expressing membrane-GFP. Rendered confocal sections were projected with total 12 □m thickness. Time-lapse images were taken every 5 min for 3 h.

Title: Supplementary Movie 8: Dynamic filopodia-like protrusions formed by mesoderm cells contact the adjacent endoderm

Description: Mesodermal cells were imaged at high resolution at the interface between the mesoderm and the endoderm in wild-type E7.5 embryos expressing membrane-GFP. Rendered confocal sagittal sections were projected with a total of 6 □m thickness. Time-lapse images were taken every 5 min for 1 h.

Title: Supplementary Movie 9: In mutant embryos the space between the mesoderm and the adjacent epithelia is absent

Description: Mutant mesodermal wing cells were densely packed. Imaging was done in optical sagittal sections of the mesodermal wings in a E7.5 mutant *β-Pix□Epi* embryo expressing membrane-GFP. Rendered confocal sections were projected with total 6 □m thickness. Time-lapse images were taken every 5 min for 2 h.

Title: Supplementary Movie 10: Protrusions in a wild-type mesoderm cell are thin, filopodia-like and highly dynamic

Description: The membrane-GFP nascent mesoderm cell was imaged from the embryo's lateral side (sagittal optical sections) close to the visceral endoderm in an E7.5 wild-type embryo with mosaic

expression of membrane-GFP, under control of EIIA-Cre. Rendered confocal sections were projected with total 8 μm thickness. Time-lapse images were taken every 5 min for 1 h 15 min.

Title: Supplementary Movie 11: Protrusions in a mutant mesoderm cell are wide and short

Description: The membrane-GFP nascent mesoderm mutant cell was imaged sagitally adjacent to the anterior visceral endoderm marked by cytoplasmic Hex-GFP signal in an E7.5 mutant *β-PixΔEpi* embryo with mosaic expression of membrane-GFP. Rendered confocal sections were projected with total 8 μm thickness. Time-lapse images were taken every 5 min for 35 min.

Title: Supplementary Movie 12: Wild-type mesodermal wing cells have long filopodia-like protrusions extending from the cell surface

Description: High-resolution surfaces of the individual mesoderm cells were created from the membrane-GFP signal from sagittal images of a live E7.5 wild-type embryo. 3D reconstructions of all individual cells were overlaid on the rendered image of membrane-GFP embryo and rotated 360° to reveal a full view of the morphology and positions of mesoderm cells.

Title: Supplementary Movie 13: Mutant mesodermal wing cells have smaller and shorter protrusions

Description: High-resolution surfaces of the individual mesoderm cells were created from the membrane-GFP signal from sagittal images of a live E7.5 mutant *β-PixΔEpi* embryo. 3D reconstructions of all individual cells were overlaid on the surface the mutant embryo (created using membrane-GFP signal and labeled semi-transparent cyan) and rotated 360° to reveal a full view of the morphology and positions of mutant mesodermal wing cells.

Title: Supplementary Movie 14: Wild-type mesodermal wing cells migrate unidirectionally

Description: 3D reconstructions of mesodermal wings' cells were rendered from sagittal confocal images of live membrane-GFP E7.5 embryos. The semi-transparent surfaces of selected mesoderm cells with their 3D displacement vectors (white cylindrical arrows) and their migration tracks (time color-coded) were overlaid on the rendered image of a membrane-GFP embryo. Unidirectional migration of mesodermal wings' cells was manifested in the alignment of displacement vectors and migration tracks within multiple rows of cells. Time-lapse images were taken every 5 min for 1 h.

Title: Supplementary Movie 15: Mutant mesodermal wing cells migrate randomly

Description: 3D reconstructions of mesodermal wings' cells were rendered from sagittal confocal images of live membrane-GFP E7.5 *β-Pix□Epi* mutant embryos. The semi-transparent surfaces of selected mesoderm cells with their 3D displacement vectors (white cylindrical arrows) and their migration tracks (time color-coded) were overlaid on the rendered image of a membrane-GFP mutant embryo. Random migration of mesoderm mutant cells was observed as misalignment of displacement vectors, opposing directions, and looping migration tracks. Time-lapse images were taken every 5 min for 1 h.

Title: Supplementary Movie 16: Wild-type cells align their longest axes
Description: Cell surfaces of the individual mesoderm cells were created from the membrane-GFP signal from sagittal images of a live wild-type E7.5 embryo. The longest axis was visualized over time. At least 5-6 cell rows in wild-type nascent mesoderm in the sagittal section were aligned. This alignment is maintained over time. Time-lapse images were taken every 5 min for 1 h.

Title: Supplementary Movie 17: Mutant cells fail to align their longest axes
Description: Cell surfaces of the mutant individual mesoderm cells were created from the membrane-GFP signal from sagittal images of a live E7.5 mutant *β-Pix□Epi* embryo. The longest axis was visualized over time and revealed misalignment of the axes in the mutant embryo. The orientation of the axes was highly dynamic and variable. Time-lapse images were taken every 5 min for 1 h.

Title: Supplementary Movie 18: The position of protrusions in wild-type mesoderm cells is aligned with the direction of cell movement
Description: Alignment between the direction of the dominant protrusion and tissue flow was maintained over time in a wild-type embryo. Maps of selected mesoderm cells with highlighted magenta protrusions, longest axes (line) and centroids (sphere), their surfaces with projected 3D displacement vectors (white arrows) on the background of the membrane-GFP embryo were created from sagittal sections of the live E7.5 wild-type embryo. Time-lapse images were taken every 5 min for 1 h.

Title: Supplementary Movie 19: The position of protrusions in mutant mesoderm cells is not aligned with the direction of cell movement
Description: Lost alignment between the direction of the dominant protrusion and tissue flow was observed in a mutant *β-Pix□Epi* embryo. Maps of selected mesoderm cells with highlighted magenta protrusions, longest axes (line) and centroids (sphere), their surfaces with projected 3D displacement

vectors (white arrows) on the background of the membrane-GFP embryo were created from sagittal sections of the live E7.5 mutant *β-Pix□Epi* embryo. Time-lapse images were taken every 5 min for 1 h.
